# Intensity-dependent tACS entrainment effects in a cortical microcircuit: a computational study

**DOI:** 10.1038/s41598-026-37594-9

**Published:** 2026-01-31

**Authors:** Kyeongseop Park, Hyeyeon Chung, Hyeon Seo, Sung Chan Jun

**Affiliations:** 1 School of Electrical Engineering and Computer Science, Gwangju Institute of Science & Technology, 123 Cheomdangwagi-ro, Buk-gu, Gwangju, 61005 Republic of Korea; 2 Department of Computer Science and Engineering, Gyeongsang National University, 501 Jinju-daero, Jinju-si, Gyeongsangnam-do 52828 Republic of Korea; 3 Department of AI Convergence, Gwangju Institute of Science & Technology, 123 Cheomdangwagi-ro, Buk-gu, 61005 Gwangju, Republic of Korea

**Keywords:** Biological techniques, Engineering, Neuroscience

## Abstract

Transcranial alternating current stimulation (tACS) is a promising noninvasive technique for modulating disrupted neural oscillations in psychiatric disorders and enhancing cognitive functions. However, its efficacy remains debated, partly because neuronal morphology and other microscopic factors critically affect the response to external electric fields. To address this issue and investigate cellular- and network-level mechanisms underlying tACS-induced neural entrainment, we developed a cortical microcircuit model integrating realistic neuronal morphologies, synaptic connectivity, and intrinsic oscillatory dynamics. Using the NEURON simulation environment, we constructed a microcircuit comprising five distinct biophysical cortical neuron models. Neural responses to a range of tACS intensities were assessed with metrics based on cross-correlation, phase coherence, and phase-locking value. While tACS modulates spike timing without significantly altering firing rates, pyramidal neurons are particularly sensitive to external fields compared to interneurons. In addition, tACS can either disrupt or enhance synchronization depending on the endogenous oscillation and stimulation intensity. Our computational study reveals that tACS effects arise from a complex interplay between intrinsic neuronal properties and network dynamics. These findings underscore the importance of neuronal morphology in determining tACS responses and provide insights that may help optimize stimulation parameters for precise neuromodulation in both clinical and research settings.

**Supplementary Information:**

The online version contains supplementary material available at 10.1038/s41598-026-37594-9.

## Introduction

Transcranial alternating current stimulation (tACS) has emerged as a promising noninvasive neuromodulation technique with considerable potential for modulating neural oscillations, enhancing cognitive functions, and alleviating symptoms of psychiatric disorders^1^. By applying weak alternating currents to the brain, tACS generates an electric field (EF) that influences neuronal activity. In recent years, numerous in vivo and in vitro studies using nonhuman animals have examined how tACS modulates neural oscillations, thereby introducing the concept of neural entrainment^2–6^. Neural entrainment refers to the synchronization of neuronal spiking activity with an external EF, such that spikes preferentially occur at specific phases of the stimulation waveform^2–6^. Importantly, the entrainment effects have been shown to increase with higher tACS amplitudes and to be frequency-dependent^1,2,4,7,8^. For example, an in vivo study in ferrets demonstrated the Arnold tongue phenomenon, wherein stimulation frequencies that are close to endogenous oscillations result in stronger entrainment, and increasing tACS intensity broadens the effective frequency range^2^.

Despite increasing interest in tACS, its effectiveness in inducing robust neural entrainment remains controversial^9,10^. At the microscopic level, variability in neuronal responses to the external EF can be attributed to differences in cell morphology and biophysical properties^11–20^. Even among neurons of the same type, subtle morphological variations may alter membrane polarization and modulate spike timing^12,16–19^. Studies have identified several morphological factors that influence neuronal activation in response to EFs, including dendritic orientation relative to the EF^13,15^, the distance between the soma and apical dendrites, and the complexity of dendritic arborization^11,16,17^. Moreover, computational studies incorporating realistic axonal models suggest that neurons may exhibit a preferred EF direction, leading to stronger activation as a result of their complex arborized structures^18–20^. These findings underscore the complexity of tACS mechanisms and suggest that intrinsic morphological differences are key contributors to the observed variability in neural responses.

To address these uncertainties and explore stimulation parameters beyond experimental constraints, computational modeling serves as an invaluable bridge between theory and experiment. Some studies have employed single-neuron models with realistic morphologies to explore the relation between neuronal structure and EF sensitivity^12,17–21^. However, evidence from both in vivo and in vitro experiments indicates that neural networks exhibit greater sensitivity to EFs compared with isolated neurons^2,22,23^. Given that brain oscillations arise from the interplay between excitatory and inhibitory synaptic inputs, with somatic polarization resulting from the summation of spatially distributed postsynaptic potentials, incorporating synaptic connectivity into computational models is essential for a comprehensive understanding of tACS effects^24^.

Researchers have investigated tACS effects using both neuron mass models^22,25–27^ and biophysical network models with simplified neuronal morphologies^2,7,28,29^. However, neuron mass models do not capture individual neuronal responses^7^, and simplified neuron models often lack the morphological detail needed to accurately reflect interactions with EFs^11,12,18^. To our knowledge, only one study has constructed a morphologically realistic neuronal network to explore the effects of electrical stimulation^30^. That columnar cortical model successfully replicated neural responses to intracortical microstimulation, including short-latency excitation, prolonged inhibition, and rebound excitation, but modeled synaptic inputs via randomized Poisson spike trains without incorporating direct synaptic connectivity. Moreover, its primary aim was to elucidate cellular and synaptic mechanisms underlying intracortical microstimulation, a modality that differs from the tACS approach examined here.

In this study, we aim to advance the understanding of synaptic interactions in response to tACS by constructing a synaptically connected cortical microcircuit comprising a small yet representative set of morphologically detailed cortical neuron models. This simplified columnar model consisting of three pyramidal cells and two interneurons strikes an optimal balance between computational efficiency and model complexity, thereby facilitating the interpretation of the functional roles of distinct neuronal subtypes. By incorporating direct synaptic connections among realistic neurons, our model uniquely captures the interplay between individual neuronal morphology and network dynamics under tACS.

Incorporating diverse cell types into cortical microcircuit models is critical for elucidating how pyramidal cells and interneurons transmit information across cortical layers^31^. Pyramidal cells, which predominate in the mammalian cerebral cortex^32^, are central to higher cognitive functions such as sensorimotor control^33^. These cells integrate synaptic inputs within the network, facilitate excitatory signaling across multiple layers^34,35^, and establish long-range projections that coordinate sensory, motor, and cognitive processes across distant brain regions^36–38^. In contrast, interneurons are crucial for regulating excitatory activity and fine-tuning synaptic integration, thereby contributing to efficient information processing^34,39^. Several studies have highlighted the role of interneurons in maintaining the excitation–inhibition balance during neocortical oscillatory processes^40–43^. In our cortical microcircuit, we incorporated two key interneuron types—a layer 1 neurogliaform cell (slow-spiking) and a layer 4 large basket cell (fast-spiking)—which are essential for network homeostasis and the coordination of brain rhythms^42,44,45^. Moreover, dysfunction in these interneurons has been linked to cortical abnormalities and altered oscillatory activity in various psychiatric disorders^40,41,46,47^.

Through this simplified cortical microcircuit with realistic morphologies and synaptic connections, we investigated how tACS modulates neural activity at both cellular and network levels. Our computational approach yielded three key findings. First, our model reproduced in vivo and in vitro observations indicating that tACS can drive phase locking without significantly altering firing rates, thus reinforcing its potential as a neuromodulatory tool^4,48^. Second, the simulations revealed that under specific endogenous oscillatory conditions, low-intensity tACS induces desynchronization, while higher intensities enhance neural entrainment, findings that align with recent studies^7,49^. Third, our results suggest that tACS effects on neural entrainment are mediated by an interplay between morphology-driven EF sensitivity and network dynamics, with certain neuron types being more influenced by EF sensitivity and others by network connectivity. Overall, our microcircuit model provides insights into the cellular-level responses to tACS across different intensities, which may improve the predictability of neuronal responses to stimulation.

## Results

### Microcircuit architecture and neural responses to tACS

**Fig. 1:**
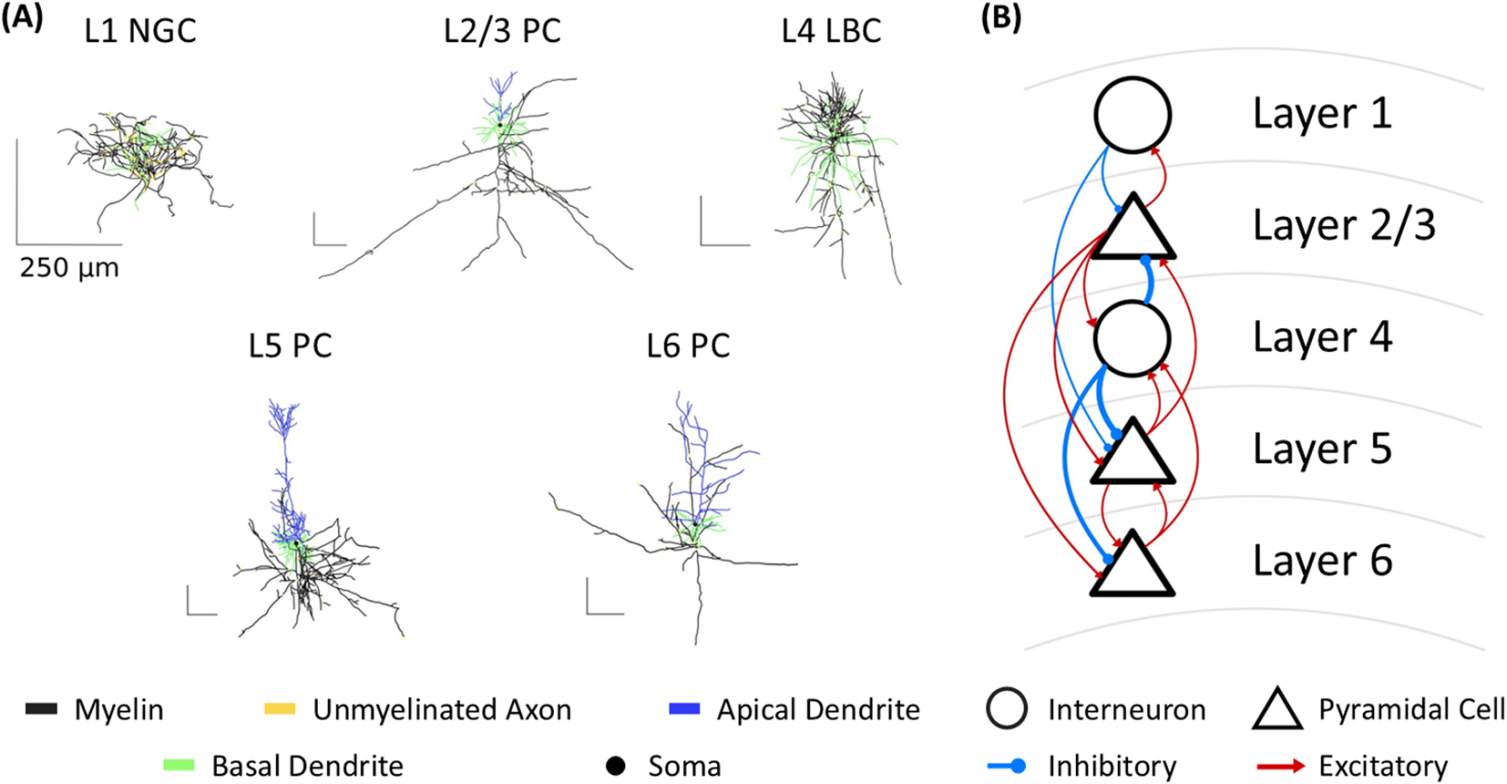
Overview of the cortical microcircuit model. (**A**) Morphologies of biophysical human cortical neuron models, including a Layer 1 neurogliaform cell (L1 NGC), Layer 2/3 pyramidal cell (L2/3 PC), Layer 4 large basket cell (L4 LBC), Layer 5 pyramidal cell (L5 PC), and Layer 6 pyramidal cell (L6 PC). (**B**) Schematic representation of the microcircuit model, illustrating inhibitory (blue) and excitatory (red) synaptic connections. Line width denotes synaptic weight between the connected cells.

To provide a mechanistic foundation for analyzing neural entrainment under tACS, we constructed a biophysically realistic cortical microcircuit consisting of five morphologically detailed neuron models spanning layers 1 through 6^18^ (Fig. 1A, Supplementary Figs. S1 and S2). Specifically, the microcircuit included layer 1 neurogliaform cell (L1 NGC), layer 2/3 pyramidal cell (L2/3 PC), layer 4 large basket cell (L4 LBC), layer 5 thick-tufted pyramidal cell (L5 PC), and layer 6 tufted pyramidal cell (L6 PC). Synaptic connectivity among these neuron models was established based on the Blue Brain Project^34,35,50^, utilizing experimental data on synaptic weights and time constants for excitatory and inhibitory synaptic connections (Fig. 1B; for more details, refer to Methods-Modeling Synaptic Inputs and Synaptic Connections).

Endogenous oscillatory activity was simulated by applying fully randomized Poisson-driven synaptic inputs to each neuron model^7,12^, with 20 independent simulations conducted for each tACS amplitude. To emulate in vivo conditions, we targeted both alpha-band and theta-band oscillations. For the alpha-band condition (with an exogenous frequency of 10 Hz), input firing rates were calibrated to 5 Hz, 10 Hz, 30 Hz, 10 Hz, and 10 Hz for L1 NGC, L2/3 PC, L4 LBC, L5 PC, and L6 PC, respectively. Different firing rates across neuron types were set to reflect the firing rates observed in vivo^42,51–53^. For the theta-band condition (exogenous frequency of 5 Hz), the firing rates were uniformly set to approximately 5 Hz across all neuron types. Uniform firing rates minimized the influence of intrinsic firing rate differences on the neural entrainment effect, in line with the Arnold tongue phenomenon^2,7^. The power spectral density was computed from the local field potentials (LFPs) obtained from 15-s 0 mA simulation (no stimulation) trials under alpha and theta oscillations. The resulting power spectral densities showed a spectral peak at the expected frequencies, with 10 Hz for alpha endogenous oscillations and 5 Hz for theta endogenous oscillations (see Supplementary Fig. S3).

**Fig. 2:**
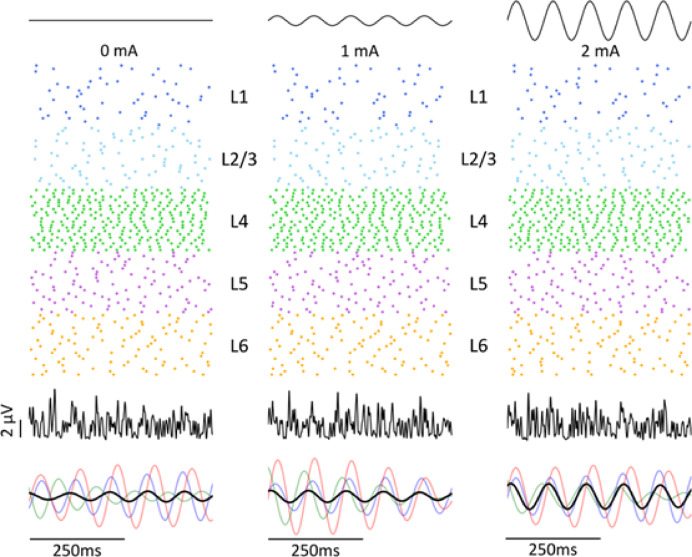
Cellular and network response to tACS. Top panel: Waveforms of 10 Hz tACS at 0 mA, 1 mA, and 2 mA from 7500 to 8000 ms. Middle panel: Raster plot of neuron spiking activity across Layer 1 to Layer 6 in response to tACS. Each line represents a single simulation trial from 7500 to 8000 ms. Each tACS condition was simulated 20 times with varying Poisson inputs to account for ongoing spiking activity. Bottom panel: Average raw LFP signals across 20 simulations from 7500 to 8000 ms. The plot below shows filtered LFP signals where the black trace represents the averaged filtered LFP across 20 simulations. Signals were processed using a 2nd order Butterworth bandpass filter with 9 and 11 Hz cutoff frequencies. The filtered LFP signals at 0 mA, 1 mA, and 2 mA were set to the same arbitrary unit scale. Light red, green, and blue traces denote a single filtered LFP in a single simulation trial, with one neuron selected per layer.

As shown in Fig. 2, raster plots provide a preliminary visualization of how tACS modulates neuronal activity, with pyramidal neurons increasingly firing near the rising phase of the waveform as the stimulation intensity increases. In addition, LFP traces from different neurons exhibit increasing temporal similarity at higher amplitudes, which may reflect enhanced network-level synchrony under tACS. Detailed analyses of these tACS-induced neural entrainment effects are presented in subsequent sections of the Results.

### Cross-correlation analysis of local field potentials

**Fig. 3:**
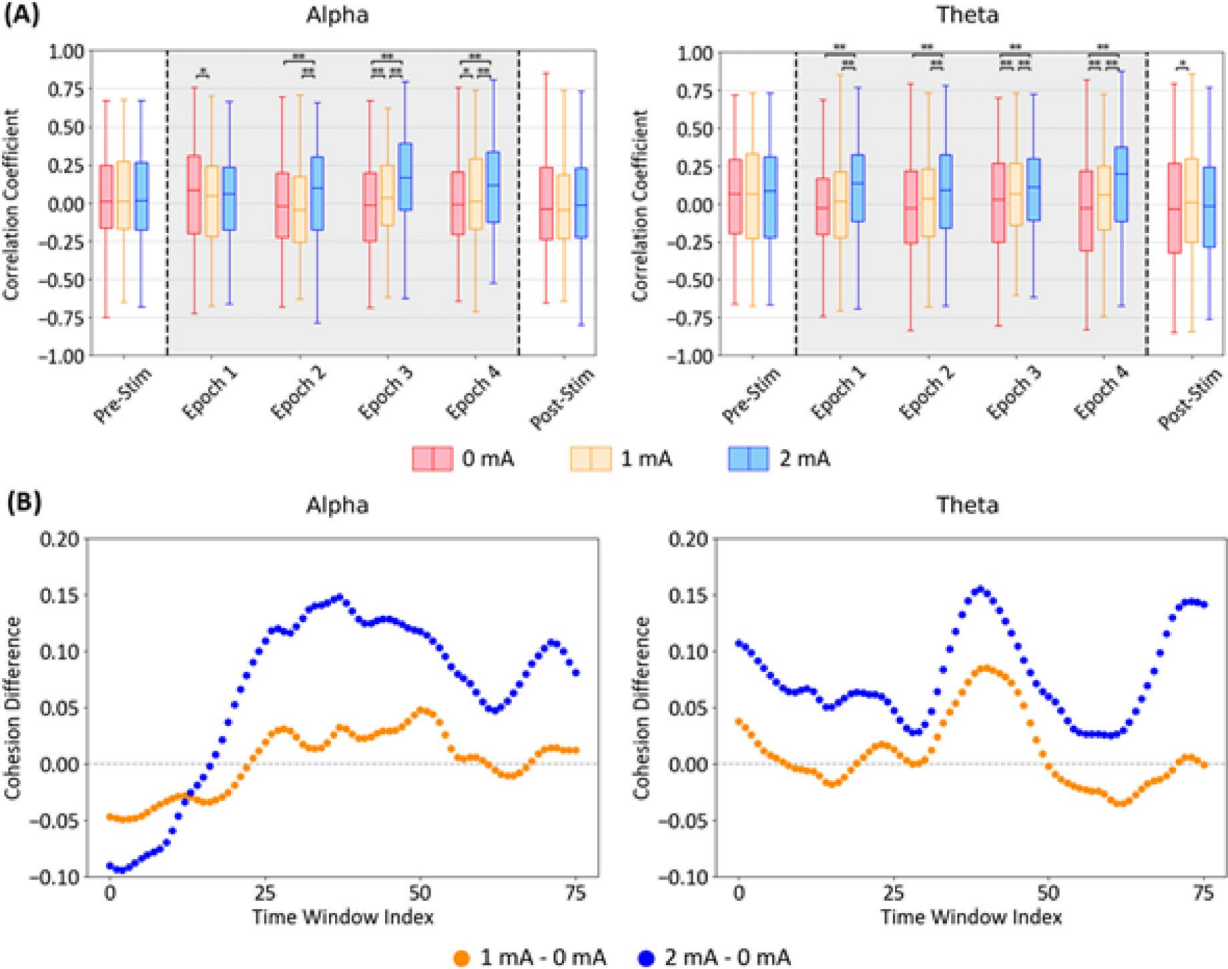
Neural entrainment across tACS intensities at the network level. (**A**) Box plots of the pairwise cross-correlation coefficients of LFPs in alpha (left) and theta (right) frequency bands over a 2.5-s period under three simulation conditions: 0 mA (red), 1 mA (yellow), and 2 mA tACS (blue). Gray shading indicates the duration of active tACS. Statistical significance between tACS conditions was assessed using the Wilcoxon signed-rank test, with significance levels denoted as * *p* < 0.05 and ** *p* < 0.005. (**B**) Time evolution of averaged phase cohesion difference between LFPs and the tACS waveform in the alpha (left) and theta (right) frequency bands during active tACS simulation. The plotted values represent the difference in phase cohesion between 0 mA and the stimulated conditions (1 mA, orange; 2 mA, blue) across time windows. A positive difference indicates stronger phase alignment to the tACS waveform compared to the 0 mA baseline condition.

Pairwise cross-correlation coefficients were computed from 20 independent LFP signals recorded under three tACS conditions (0 mA, 1 mA, and 2 mA) for both alpha and theta oscillations (Fig. 3A). As each simulation trial lasts 15 s, consisting of 2.5-s pre-stimulation, 10-s active tACS, and 2.5-s post-stimulation, the cross-correlation was computed in 2.5-s segments. Each pairwise comparison resulted in six cross-correlation values corresponding to the pre-stimulation phase, four active tACS epochs, and post-stimulation phase. During the pre-stimulation period and in the post-stimulation phase of alpha oscillations, no statistically significant differences were observed among the tACS conditions. For theta oscillations, however, significant differences emerged during the post-stimulation period between the 0 mA and 1 mA conditions (*p* < 0.05), with median cross-correlation coefficients of − 0.032, 0.008, and − 0.016 for 0 mA, 1 mA, and 2 mA, respectively.

During active tACS in the alpha band, cross-correlation differences were not apparent in the first 2.5-s epoch. After the first stimulation epoch, network synchronization increased relative to the baseline; the 2 mA condition showed a significant difference (*p* < 0.005), while the 1 mA condition demonstrated significant changes during the third and fourth stimulation epochs (*p* < 0.05). Complementary analysis using a sliding window phase coherence metric revealed a local peak around 2,800 ms after the stimulation onset. (Fig. 3B, Supplementary Fig. S4). Moreover, an increasing trend of phase coherence values with higher stimulation intensities remained consistent regardless of the sliding window size (Supplementary Fig. S5).

In the theta band, the neuronal response to tACS was immediate, with cross-correlation coefficients increasing significantly with higher tACS intensities (*p* < 0.005). The phase coherence analysis mirrored these results, with approximately 57% of sliding windows at 1 mA and all sliding windows at 2 mA exceeding the phase coherence observed under the baseline. These data indicate a prompt response of the neuron models to tACS, consistent with previous experimental findings^4,54^. The delayed synchronization in the alpha band may reflect the heterogeneous intrinsic firing rates among neuron models, supporting that a 10-s tACS duration is adequate to probe immediate entrainment mechanisms in our microcircuit, which does not account for synaptic plasticity.

### Effects of synaptic connections and tACS on firing rates

At the cellular level, firing rates were compared between conditions with and without synaptic connectivity during the 10-s tACS period (2.5–12.5 s). Under Poisson-driven inputs targeting alpha oscillations, single-neuron models without synaptic connections displayed mean firing rates (± standard deviation) of 4.84 ± 0.21 Hz (L1 NGC), 9.17 ± 0.19 Hz (L2/3 PC), 31.66 ± 0.30 Hz (L4 LBC), 10.00 ± 0.13 Hz (L5 PC), and 10.18 ± 0.14 Hz (L6 PC). With synaptic connectivity, firing rates shifted slightly to 5.10 ± 0.21 Hz, 9.12 ± 0.21 Hz, 31.74 ± 0.31 Hz, 9.99 ± 0.13 Hz, and 10.18 ± 0.14 Hz for L1 NGC, L2/3 PC, L4 LBC, L5 PC, and L6 PC, respectively. The interneurons (receiving only excitatory inputs) exhibited modest increases—0.26 Hz for L1 NGC and 0.08 Hz for L4 LBC—while pyramidal cells (receiving both excitatory and inhibitory inputs) showed only marginal fluctuations (e.g., a − 0.05 Hz change for L2/3 PC).

A similar pattern was observed for theta oscillations. Without synaptic connectivity, firing rates were 4.84 ± 0.21 Hz (L1 NGC), 4.34 ± 0.07 Hz (L2/3 PC), 4.52 ± 0.18 Hz (L4 LBC), 4.75 ± 0.10 Hz (L5 PC), and 5.32 ± 0.23 Hz (L6 PC). With connectivity, these rates became 5.06 ± 0.21 Hz, 4.32 ± 0.05 Hz, 4.68 ± 0.15 Hz, 4.71 ± 0.11 Hz, and 5.32 ± 0.23 Hz, respectively. In both oscillatory conditions, interneurons consistently showed increased firing rates, whereas pyramidal cells remained largely unaffected (< 0.05 Hz change).

### Relation between neural entrainment and tACS intensity

**Fig. 4:**
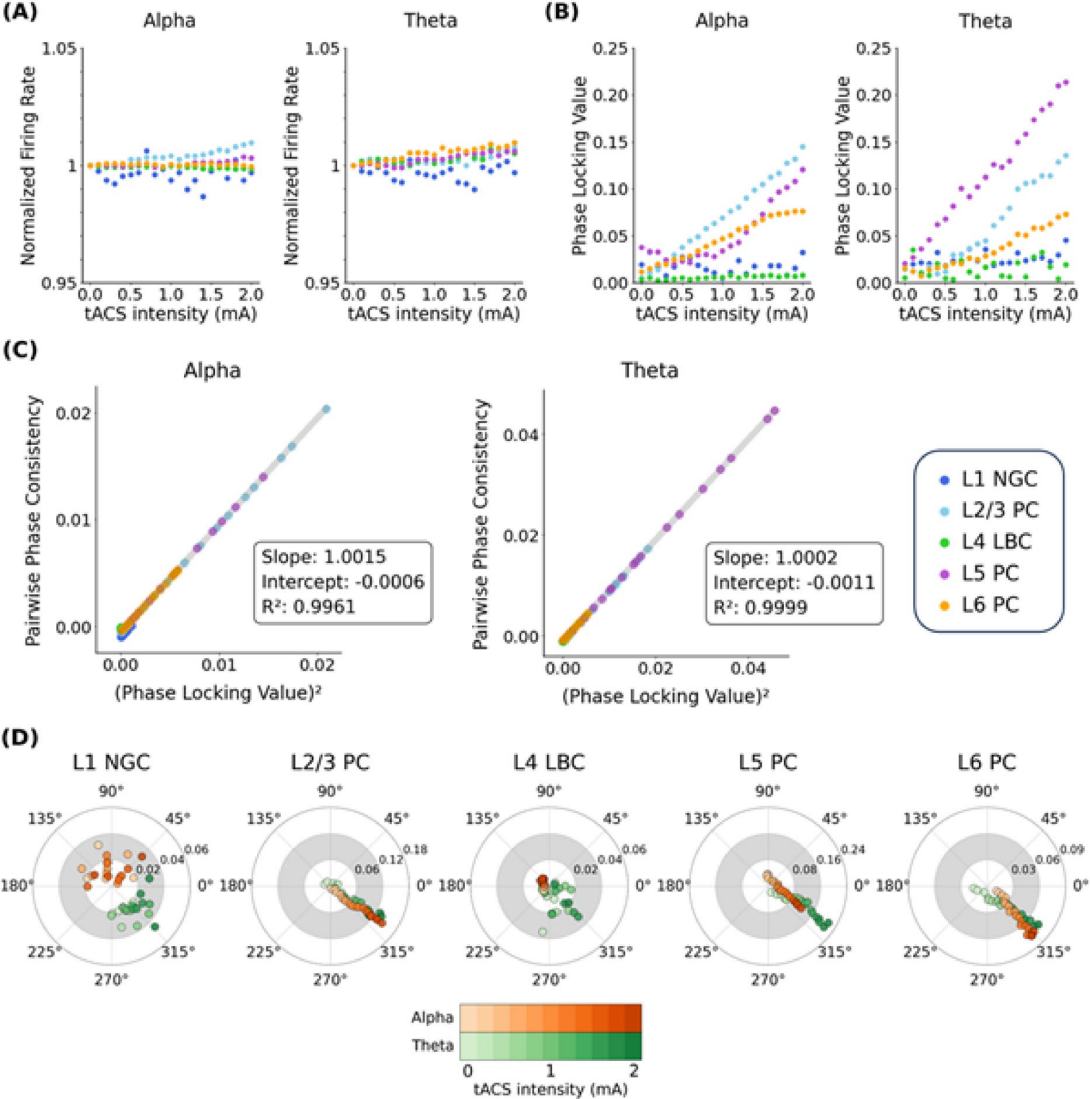
Neural entrainment across tACS intensities at the cellular level. (**A**) Normalized firing rate across different tACS intensities for alpha (left) and theta (right) endogenous oscillations. The firing rate is averaged across the 20 simulations. (**B**) PLV corresponding to tACS intensity in alpha (left) and theta (right) oscillations. (**C**) Scatter plot of squared PLV versus PPC for alpha (left) and theta (right) endogenous oscillations. Each dot represents a neuron type under a specific tACS intensity. The gray line denotes the linear regression line. (**D**) PLV and preferred phase across tACS intensity levels for alpha (orange) and theta (green) oscillations, with darker shades indicating stronger stimulation intensities. Spiking phases recorded during the tACS period from 20 independent simulations were aggregated to calculate a single PLV and preferred phase for each neuron type and tACS amplitude.

Neural entrainment was quantified by measuring the phase-locking value (PLV) and preferred phase across a range of tACS intensities for both alpha and theta oscillations (Fig. 4; Supplementary Table 1). After normalizing firing rates to the mean firing rate recorded across trials in the 0 mA baseline condition, we observed that firing rate changes remained below 1% for both oscillatory conditions (Fig. 4A), reinforcing that tACS primarily modulates spike timing rather than firing rate^4,12,48^.

Experimental studies recording single neuron activity in the cortex of awake nonhuman primates during tACS reported PLV ranging from 0.10 to 0.19 at 1–2 mA tACS for responsive neurons (Rayleigh test, *p* < 0.01)^4,49^. Responsive neurons in our cortical microcircuit at 1–2 mA s displayed PLVs ranging from 0.07 to 0.22 (Fig. 4B), which are consistent with the PLVs in experimental studies^4,49^. Additionally, pyramidal neurons with initial preferred phases differing from those observed at 2 mA exhibited a reduction in PLV at lower stimulation intensities, followed by an increase once their preferred phase shifted toward the rising phase of the tACS waveform (for PLV and preferred phase values, see Supplementary Table 1). In these neurons, PLV decreased until approximately 0.2 mA (L6 PC in theta) or 0.4 mA (L2/3 PC in theta and L5 PC in alpha) before rising with increased stimulation intensity. Conversely, neurons with already aligned preferred phases showed a continuous increase in PLV.

Across both frequency bands, pyramidal cells exhibited higher PLV values than interneurons (Fig. 4B; see also Supplementary Figs. S6 and S7). Mean PLVs over tACS intensities from 0.4 to 2.0 mA were 0.016 (L1 NGC) and 0.027 (L1 NGC) for alpha and theta oscillations, respectively, and 0.006 (L4 LBC) and 0.015 (L4 LBC) for theta oscillations. The PLV slope versus tACS intensity above 0.4 mA was 0.069, 0.065, and 0.036 in alpha oscillations and 0.082, 0.094, and 0.038 in theta oscillations for L2/3 PC, L5 PC, and L6 PC, respectively. Linear regression was computed separately for each neuron type, revealing a strong linear relation for pyramidal cells, with R^2^ values exceeding 0.9. In contrast, interneurons generally showed weak linearity (R^2^ < 0.1) except for L4 LBC in alpha oscillations (R^2^ = 0.633). Applying stimulation frequencies of 5, 8, and 12 Hz at 1 mA, which were intentionally offset from intrinsic alpha oscillations, also increased PLVs and spiking phase non-uniformity compared to the baseline (Supplementary Figs. S8 and S9). Stimulation at 8 and 12 Hz, frequencies closer to endogenous oscillations, produced stronger entrainment than the more distant 5 Hz condition, in line with the Arnold tongue phenomenon^1,2,4,7,8^.

Previous studies have noted that the PLV metric can be biased when the number of spike counts is insufficient or when comparing neurons with different spike counts^54–56^. To evaluate this potential bias, we computed the pairwise phase consistency (PPC), a sample-size–independent entrainment metric, and compared it with the squared PLV (Fig. 4C)^54^. Linear regression was performed across all neuron types for each endogenous oscillation. We observed that the squared PLV closely resembled the PPC^55^. In both the alpha and theta conditions, the slope was < 1.002, the intercept was within ± 0.002, and the R^2^ exceeded 0.99. Moreover, the PPC–PLV relationship remained consistent across different tACS intensities, with pyramidal cells exhibiting higher values than interneurons (Supplementary Fig. S10). These results indicate that the computed PLVs are not biased by limited sample size or differences in spike counts.

Polar plot analyses revealed that when endogenous and exogenous oscillations were initially out of phase, the preferred phase gradually shifted toward the tACS waveform, converging at intensities below 1 mA (Fig. 4D). This convergence was consistent across both frequency conditions, with pyramidal cells aligning toward the rising phase (311.8–329.3°). At or above 1 mA, the preferred phases of pyramidal cells stabilized at 324.0° ± 2.0° (L2/3 PC), 335.3° ± 7.0° (L5 PC), and 313.4° ± 2.0° (L6 PC) in alpha, and 327.1° ± 3.8°, 330.5° ± 4.5°, and 319.6° ± 1.8° in theta oscillations. In contrast, interneurons showed greater variability, with preferred phases of 75.8° ± 48.5° and 138.8° ± 8.3° in alpha, and 324.4° ± 19.6° and 314.1° ± 38.5° in theta oscillations for L1 NGC and L4 LBC, respectively.

Further quantification of phase alignment was achieved by converting polar coordinates (θ, r) into Cartesian coordinates (x = r cos θ, y = r sin θ) and performing linear regression. For pyramidal cells, the R^2^ values exceeded 0.85 at tACS intensities of 1 mA and above, indicating a strong linear relation between phase alignment and stimulation intensity. By contrast, interneurons produced R^2^ values below 0.4, reflecting their higher phase variability (Fig. 4D).

### Neural entrainment effects modulated by synaptic connectivity

**Fig. 5:**
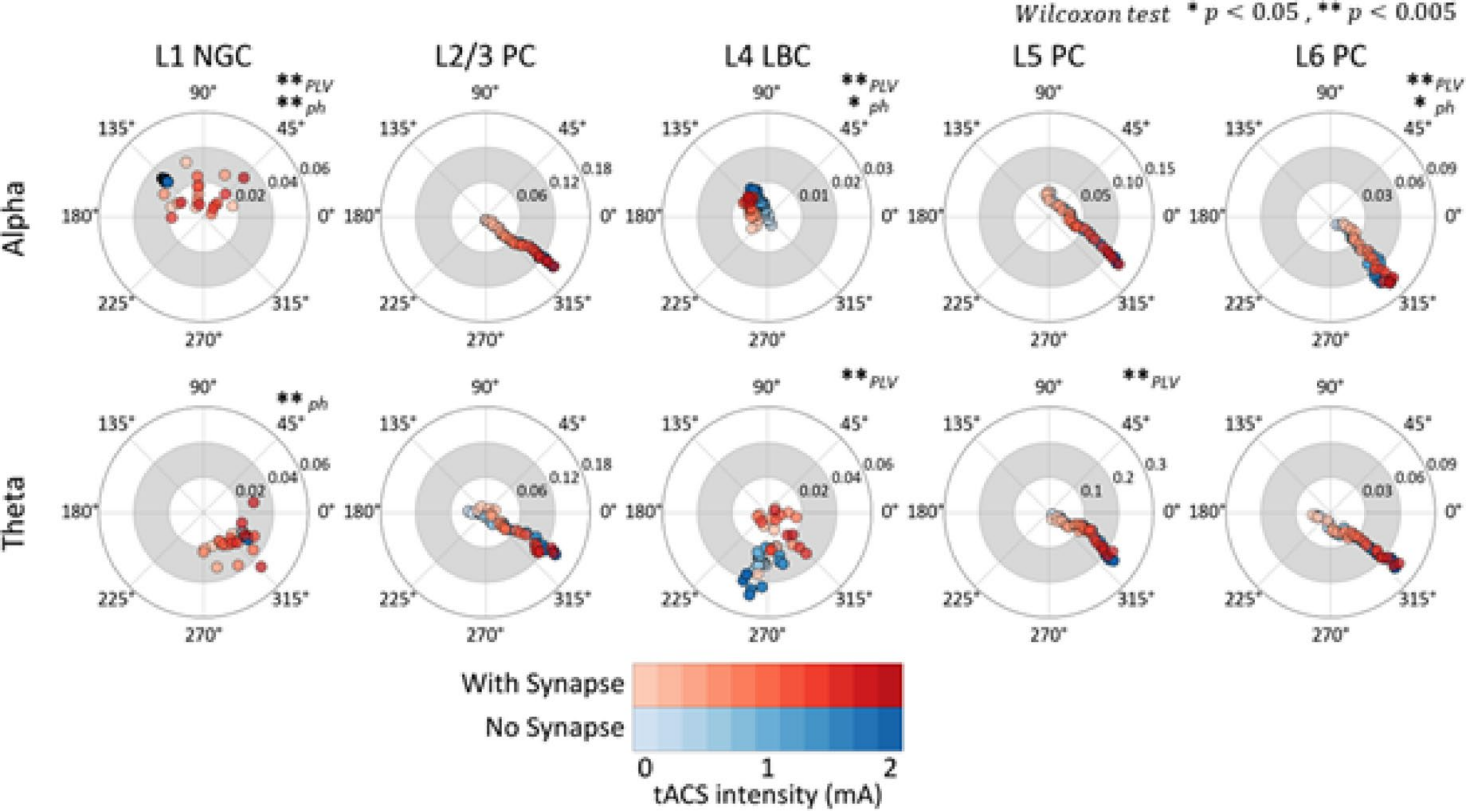
Effects of synaptic connectivity in response to tACS. Comparison of PLV and preferred phase across tACS intensities in alpha and theta oscillations between a synaptically connected microcircuit (red) and a synaptically disconnected microcircuit receiving only Poisson synaptic inputs (blue). Statistical significance of PLV and preferred phase (ph) differences was assessed using the Wilcoxon signed-rank test, with significance levels indicated as * *p* < 0.05, ** *p* < 0.005 at the upper right of the corresponding polar plot.

To assess the role of synaptic connectivity in tACS-induced neural entrainment, we compared PLV and preferred phase metrics between conditions with full synaptic connectivity and conditions with only Poisson-driven inputs (i.e., disconnected state) (Fig. 5; Supplementary Figs. S11 and S12; Supplementary Table 1). In L1 NGC, the introduction of synaptic connections produced a marked increase in phase variability; the circular standard deviation of preferred phases increased from 0.78° (alpha) and 1.26° (theta) in the disconnected state to 48.52° (alpha) and 24.43° (theta) with connectivity. Similarly, L4 LBC exhibited significant shifts in PLV distributions (*p* < 0.005) and an increased preferred phase in the alpha condition (*p* < 0.05) upon the inclusion of synaptic connections.

According to the Wilcoxon test in pyramidal cells, differences emerged for L6 PC in alpha oscillations and L5 PC in theta oscillations. For L6 PC, the PLV slope in alpha oscillations was 0.034 in the disconnected state versus 0.036 when connected, accompanied by a preferred phase shift from 315.0° ± 5.7° to 318.1° ± 7.2°. In L5 PC under theta oscillations, synaptic connectivity reduced the PLV slope from 0.099 to 0.095. Overall, interneurons displayed more pronounced differences in both PLV and preferred phase across tACS intensities when comparing connected and disconnected conditions, highlighting the dominant influence of synaptic inputs relative to direct tACS intensity in shaping interneuron responses.

## Discussion

In this study, we developed a cortical microcircuit model incorporating realistic neuronal morphologies and synaptic connectivity to investigate tACS-induced neural entrainment at both the network and cellular levels. At the network level, our cross-correlation and phase coherence analyses revealed that LFPs followed the tACS waveform when stimulation was applied (Fig. 3). These observations align with previous experimental^1,2,4,54^ and computational studies^7,12^ and indicate that neuronal networks quickly respond to tACS. These values stabilized within the 10-s stimulation window, suggesting that the microcircuit model had settled into a stable entrainment state. Notably, when intrinsic firing rates were matched across neurons as in theta oscillations, LFP synchronization increased rapidly, whereas in the alpha band—where intrinsic firing rates were heterogeneous—synchronization emerged more gradually. Such differences suggest that the degree and speed of tACS-induced entrainment depend on endogenous network dynamics; networks already tuned to a specific frequency synchronize more readily than those with diverse oscillatory patterns^1,2,7^. The absence of significant changes in post-stimulation cross-correlation across tACS intensities further supports the hypothesis that lasting aftereffects in human tACS studies^57–59^ might require plasticity-dependent mechanisms^60,61^ that our model does not address. Although our framework does not account for long term mechanisms of tACS, the results show that 15-s trial length was sufficient to probe the immediate entrainment effects in the microcircuit.

At the cellular level, our analyses confirm that tACS modulates spike timing without substantially altering overall firing rates (Fig. 4A), consistent with both experimental^4,48,49,54^ and computational^7,12^ studies. Pyramidal neurons exhibited a clear, linear relation between phase-locking value (PLV) and tACS intensity above 0.4 mA, whereas interneurons showed more variable responses (Fig. 4B). The lack of increased PLV for interneurons across tACS intensities in the alpha-band condition may be attributed to a difference between their intrinsic firing rates and the tACS frequency. However, the outcome in the theta-band condition was similar to the outcome in the alpha-band, even though the tACS frequency and the firing rate were matched. These findings echo previous reports from single-neuron models^12–14^, suggesting that the elongated and directionally oriented dendritic trees of pyramidal cells render them more sensitive to applied electric fields. In contrast, interneurons, which lack these morphological features (see Supplementary Fig. S1), demonstrate lower and less consistent entrainment. Consequently, dendritic morphology in terms of length and spatial orientation emerges as a critical determinant of a neuron’s sensitivity to external electric fields.

Our results further reveal that tACS can either weaken or enhance neural entrainment depending on the interplay between endogenous oscillations and stimulation intensity (Fig. 4D). When endogenous spike timing is not phase-coherent with the external waveform, weak tACS can transiently disrupt ongoing activity, a phenomenon supported by recent *in vivo*^49^ and simplified network studies^7^. However, at intensities of 1 mA and above, the external stimulation dominates, driving a stable entrainment pattern in which the preferred phase of pyramidal neurons converges toward the rising anodic phase (311.8°–329.3°) regardless of the stimulation frequency. Regarding the clinical implications of PLV, rodent studies suggest that an increase of approximately 0.1 in PLV relative to the baseline can enhance working memory^62^ and reward prediction^63^. Conversely, excessive synchrony has been associated with psychiatric disorders^48^ such as Parkinson’s disease, where patients exhibit PLVs of up to 0.3^64^. Therefore, these results underscore the need to optimize stimulation parameters such as intensity and phase alignment for therapeutic purposes, whether the goal is to counteract pathological synchrony^64–66^ or enhance beneficial synchrony associated with cognitive functions^49,67,68^.

To further explore how network interactions shape tACS effects, we compared entrainment metrics under connected and disconnected conditions (Fig. 5). Although pyramidal neurons showed only modest differences, the observed shifts in preferred phase and variations in PLV across neuron models were statistically significant, consistent with recent computational studies on the effects of excitatory and inhibitory synaptic inputs on entrainment^7^. For instance, increased excitatory synaptic strength in pyramidal cells has been shown to induce a forward phase shift—i.e., moving the preferred phase closer to the peak of the stimulus waveform—and to elevate PLV, while enhanced inhibitory strength tends to reduce PLV. Our data reflect this trend; specifically, L6 pyramidal cells in alpha oscillations demonstrated both an increase in PLV and a forward phase shift, whereas L5 pyramidal cells in theta oscillations exhibited a reduction in PLV. Moreover, in alpha oscillations, interneurons displayed a preferred phase opposite to that of pyramidal neurons, possibly reinforcing excitatory inputs and attenuating inhibitory influences on L6 pyramidal cells (Fig. 4D). In theta oscillations, however, interneurons aligned their preferred phase with the anodic phase of the tACS waveform, similar to pyramidal cells, suggesting that inhibitory inputs to L5 pyramidal cells, derived from L1 NGC and L4 LBC, may contribute to the reduced PLV observed under theta conditions. Collectively, these findings indicate that while direct stimulation parameters primarily drive neural responses to tACS, network-level interactions and synaptic connectivity substantially modulate entrainment outcomes.

Interneurons showed greater differences between connected and disconnected states, with higher phase variability and less consistent phase locking across tACS intensities than the more robust entrainment observed in pyramidal neurons. In the synaptically disconnected condition, interneurons exhibited negligible phase locking to tACS, with only a minimal increase in PLV across stimulation intensities (Fig. 5). For example, L1 NGC maintained stable preferred phases ranging from 135 to 138° in the alpha condition and from 329° to 334° in the theta condition (see Supplementary Table 1), suggesting that their preferred phase was largely determined by the initial Poisson-driven inputs. When synaptic connections were included, interneurons at higher tACS intensities received excitatory inputs during the rising (anodic) phase of the waveform. Because interneurons displayed low PLV compared with pyramidal cells—indicating an almost uniform phase distribution (Supplementary Figs. S6 and S7)—these inputs likely drove shifts in their preferred phase, producing variability across tACS intensities in the connected microcircuit. These results indicate that interneurons are influenced by tACS primarily through synaptic interactions rather than direct EF effects, highlighting how stimulation parameters, network dynamics, and neuronal morphology together shape neural entrainment.

Despite reproducing key experimental findings and elucidating underlying mechanisms of tACS, our microcircuit model has several limitations. First, the use of realistic neuronal morphologies greatly increases computational demands, limiting simulations to 15-s durations. In addition, static synaptic parameters were employed, precluding investigation of dynamic synaptic plasticity. An experimental study examined that a tACS duration of 6 min in healthy human participants did not lead to lasting aftereffects, and durations of 20 min or more are required to observe the effects^8^. They also stated that NMDA-mediated synaptic plasticity mainly influences the temporal phase precession, i.e., the phenomenon of gradual shifts of the neuron’s spiking phase relative to the LFP^8^. Thus, extensively longer simulations would be necessary to assess potential plasticity-dependent aftereffects, which recent studies suggest are critical to sustained tACS effects^1,8,60,61^. Future work incorporating spike-timing-dependent plasticity could shed light on the mechanisms underlying long-term tACS effects.

Finally, our simplified microcircuit, comprising only five neuron models excluding diverse types of interneuron and horizontal inter-columnar interactions, does not fully capture the complexity of the cerebral cortex. The microcircuit contained a total of 7,194 segments, requiring substantially greater computational resources than neuron models with simplified morphologies. Incorporating a more diverse population of neuron models into the current framework would further increase computation time beyond our present resources. Future work implementing larger-scale models with more accurate cell-type distributions and intercolumnar connectivity will be essential to enhance the physiological relevance of tACS-induced effects.

The primary objective of our investigation, however, was to examine how neural entrainment is shaped by morphology and synaptic connections. While previous studies have focused on either single-neuron models ^12,17–21^ or large-scale network models^7,22,25–29^, our framework integrates both elements within a minimal microcircuit. By including both morphological and synaptic factors, the model demonstrated how pyramidal cells and interneurons respond differently to tACS at the microcircuit level. Even at this scale, pyramidal neurons exhibited high sensitivity to external electric fields due to their distinctive morphology, underscoring the critical role of neuronal structure in shaping tACS responses. Thus, although our model does not fully represent the network dynamics of the real cortex, our investigation provides a step toward bridging single-neuron and large-scale network models. These findings lay the groundwork for future studies to develop multi-scale models that connect cellular, network, and whole-brain dynamics, thereby optimizing electrode montages and stimulation protocols for improved efficacy^19^.

In summary, we have developed a cortical microcircuit model that incorporates detailed neuronal morphologies and synaptic connectivity to examine tACS-induced neural entrainment at both the network and cellular levels. Our findings demonstrate that tACS rapidly drives phase-locking in LFPs, with stronger stimulation leading to increasingly robust entrainment, while overall firing rates remain largely unchanged. Pyramidal neurons, with their extensive and directionally aligned dendritic trees, exhibit greater sensitivity to tACS compared to interneurons. Notably, our results reveal a dual effect of tACS: low-intensity stimulation may transiently disrupt endogenous oscillations, whereas higher intensities yield stable entrainment with convergence of the preferred phase. These insights underscore the importance of considering both neuronal morphology and network interactions in optimizing tACS protocols.

## Methods

### Realistic neuron models

We constructed a columnar cortical microcircuit incorporating five detailed, multi-compartment neuron models representing different cortical layers using morphological and physiological data from the Blue Brain Project^34^. Specifically, our models represent a Layer 1 dense arborized neurogliaform cell (L1 NGC), a Layer 2/3 pyramidal cell (L2/3 PC), a Layer 4 large basket cell (L4 LBC), a Layer 5 thick-tufted pyramidal cell (L5 PC), and a Layer 6 tufted pyramidal cell (L6 PC). Originally derived from the somatosensory cortex of a juvenile rat^34,35^, these models were subsequently modified to better represent human neuronal characteristics^18^. The modifications included refinements to dendritic branching patterns, dendritic lengths, somatic dimensions, and axonal thickness, as well as improvements in the biophysical properties of membrane ion channels^18^.

Neuronal dynamics were simulated using the NEURON v8.2 environment^69,70^. In NEURON, each neuron’s morphology is discretized into segments that serve as the computational units for calculating transmembrane potentials. Ion channels, distributed across distinct compartments—including the soma, basal and apical dendrites, and nodes of Ranvier—are positioned at the center of each segment. Based on cable theory, the membrane potential $V\left(x,t\right)$ at a segment located at position $x$ and time $t$ is given by:

$$\frac{1}{r}\frac{{\partial }^{2}V(x,t)}{\partial {x}^{2}}-c\frac{\partial V\left(x,t\right)}{\partial t}+i=\frac{1}{r}\frac{\partial {E}_{||}\left(x,t\right)}{\partial x}$$

where $r$ is the intracellular resistance, $c$ is the membrane capacitance, and $i$ represents the ionic currents through the membrane channels. ${E}_{||}\left(x,t\right)$ denotes the component of the induced electric field along the neuron’s compartment length^71^.

### Modeling synaptic inputs and synaptic connections

To investigate the effects of weak tACS on neuromodulation, synaptic inputs were introduced via a stochastic Poisson process that generates ongoing endogenous oscillations^7,12^. In our model, excitatory synaptic inputs were delivered along the basal dendrites with an average inter-spike interval of 10 ms. These inputs were applied to multiple dendritic branches at various orientations relative to the soma, ensuring a consistent spatial distribution across simulation trials. Unique seeds for the Poisson process were used to generate postsynaptic currents under different simulation conditions, as described in recent computational studies^7,12^.

The synaptic current, ${I}_{syn}$, induced by these Poisson inputs was modeled using a dual exponential function to capture biologically plausible synaptic kinetics^72,73^:

$${I}_{syn}\left(t\right)={g}_{syn}(t)(V\left(t\right)-{E}_{syn})$$

$${g}_{syn}\left(t\right)=\overline{{g }_{syn}}\frac{{\tau }_{1}{\tau }_{2}}{{\tau }_{1}-{\tau }_{2}}({e}^{-\frac{t-{t}_{s}}{{\tau }_{1}}}-{e}^{-\frac{t-{t}_{s}}{{\tau }_{2}}})$$

Here, ${g}_{syn}(t)$ is the time-varying synaptic conductance, $\overline{{g }_{syn}}$ denotes the synaptic weight, $V(t)$ is the membrane potential, ${E}_{syn}$ is the synaptic reversal potential, and ${t}_{s}$ is the time of the presynaptic spike. The constants τ₁ and τ₂ represent the rise and decay time constants of the synaptic conductance, respectively. We adopted parameter values of ${E}_{syn}$ = 0 mV, τ₁ = 2 ms, and τ₂ = 10 ms in line with previous in silico studies that produced ongoing spiking activity^7,12,74^.

Network interactions were modeled using synaptic dynamics derived from the Blue Brain Project, incorporating both excitatory (glutamatergic) and inhibitory (GABAergic) synapses^34,35,50^. Specifically, we used modules that simulate synaptic currents mediated by α-amino-3-hydroxy-5-isoxazolepropionic acid (AMPA) and N-methyl-D-aspartate (NMDA) receptors for excitatory connections, and γ-aminobutyric acid type A (GABA_A_) and type B (GABA_B_) receptors for inhibitory connections. In our model, inhibitory synapses were implemented with cell-type-specific mechanisms: the L4 LBC employed solely GABA_A_-mediated currents, whereas the L1 NGC incorporated both GABA_A_ and GABA_B_-mediated currents^44,75^. Synaptic connections were parameterized based on peak conductance, release probability, and postsynaptic kinetics, accounting for both synaptic efficacy and the availability of synaptic resources. Additionally, the spatial distribution of postsynaptic sites was calibrated using data from both the Blue Brain Project and the Neocortical Microcircuit Collaboration portal^35^ (Supplementary Fig. S2).

Synaptic signaling was implemented via the network connection (NetCon) class in NEURON^70^, which monitors presynaptic spike events and transmits corresponding synaptic currents to designated postsynaptic locations. Each synaptic connection comprised multiple individual contacts, with each NetCon object representing a single contact. In total, our microcircuit encompassed 14 synaptic connections mediated by 109 individual NetCon objects.

### Modeling endogenous oscillations

Endogenous oscillatory activity was simulated by applying stochastic Poisson-driven inputs to each neuron model. To emulate in vivo conditions, both alpha-band (8–13 Hz) and theta-band (4–7 Hz) oscillations were investigated. Alpha oscillations, which are prominent in the human brain and widely examined in studies^61,74–78^, were primarily probed in pyramidal neurons, with interneuron firing properties carefully calibrated. Specifically, the intrinsic firing rates of interneurons were set to 5 Hz for the L1 neurogliaform cell (L1 NGC) and 30 Hz for the L4 large basket cell (L4 LBC), in accordance with in vivo data^42,51–53^. This parameterization was intended to elucidate the distinct contributions of interneurons to cortical oscillatory dynamics.

For simulations involving theta oscillations—commonly associated with complex sensorimotor integration^79–81^—we employed uniform firing rates of approximately 5 Hz across all neuron types. This standardization minimized the influence of intrinsic spiking frequency, thereby isolating the effects of morphological and synaptic factors on entrainment, in accordance with the principles underlying the Arnold tongue phenomenon^1,2,4,7,8^. In line with previous work^12,52,79^, basket cells were also modeled to oscillate at theta frequencies, further ensuring consistency in baseline activity across neuron types. Thus, contrasting heterogeneous firing rates in the alpha-band condition with homogeneous firing rates in the theta-band condition allowed us to probe which factors most strongly govern neural entrainment.

### Modeling tACS

tACS modeling was implemented in the COMSOL Multiphysics (v5.2a) by simulating a three-dimensional cubic volume (2650 µm per side) encompassing the entire microcircuit. A uniform EF was generated throughout the cubic model. The anodic phase of tACS corresponded to an orientation from cortical layer 1 to layer 6. Then, neurons were integrated virtually with a cubic model, with each soma aligned parallel to the applied EF. The corresponding computed extracellular potentials were then applied to each neuronal segment via the ‘extracellular mechanism’ in NEURON^7,69,70^, thereby simulating local EF effects on neuronal dynamics.

Simulations were conducted using sinusoidal waveforms at 10 Hz for alpha oscillations and 5 Hz for theta oscillations. The tACS amplitude was varied incrementally from 0.1 to 2 mA, consistent with current amplitudes used in clinical studies^82^. Under these conditions, a tACS intensity of 1 mA produced an EF of approximately 1 mV/mm. Previous studies reported that 1 mA induces an EF lower than 1 V/m in humans and nonhuman primates^1,4,8,12^. As a uniform EF was applied in the implemented homogeneous and isotropic cube model, the simulated EF can be linearly rescaled based on the relationship between tACS intensity and EF magnitude. To capture variability in ongoing spiking activity, 20 independent simulation runs were performed for each tACS amplitude. Each simulation lasted 15 s, comprising a 2.5-s pre-stimulation period, 10 s of active tACS, and a 2.5-s post-stimulation period. The baseline refers to an entire 15-s simulation trial without tACS.

### Local field potential

Local field potentials (LFPs) were computed to assess the impact of externally applied oscillations on the microcircuit. LFPs were estimated by summing the extracellular potentials induced by individual neuronal segments, with contributions weighted according to the distance of each segment from a recording electrode. Given the high sensitivity of L5 PCs to EFs and their robust synaptic activity, L5 PCs were considered the primary contributors to the LFP signal^83,84^. Consequently, the recording electrode was positioned 20 µm from the L5 PC soma to effectively capture LFP variations.

For a given segment, the extracellular potential, ${V}_{e}$ was estimated using the line source approximation^85^:

$${V}_{e}={I}_{mem}/4\pi r\sigma$$

Here, ${I}_{mem}$ is the transmembrane current of the segment, $r$ is the distance between the segment’s midpoint and the recording electrode, and $\sigma$ is the extracellular conductivity, set at 0.3 mS/mm^7,86,87^. Since tACS effects were confined to a narrow bandwidth around the stimulation frequency, the computed LFP signal was subsequently band-pass filtered using a second-order Butterworth filter. For alpha oscillations, the filter range was set to 10 ± 1 Hz, and for theta oscillations to 5 ± 1 Hz^49^.

### Analysis of neural entrainment

Neural entrainment was evaluated at both network and cellular levels. At the network level, we performed cross-correlation analysis on 20 independent LFP signals recorded under three tACS conditions (0 mA, 1 mA, and 2 mA) for both alpha and theta oscillations. This analysis quantified the extent to which tACS influenced the timing and coordination of network activity. Higher cross-correlation values indicate more consistent temporal patterns across trials and reduced variability in the LFP signals. For each pairwise comparison, the cross-correlation was computed within a 2.5-s window, resulting six cross-correlation values corresponding to the pre-stimulation phase, four active tACS epochs, and the post-stimulation phase. The zero-lag cross-correlation, ${r}_{xy}\left(0\right)$, between two LFP signals was calculated as follows^88^:

$${r}_{xy}\left(0\right)=\frac{{\sum }_{i=1}^{N}({X}_{i}-\overline{X })({Y}_{i}-\overline{Y })}{\sqrt{{\sum }_{i=1}^{N}{\left({X}_{i}-\overline{X }\right)}^{2}{\sum }_{i=1}^{N}{\left({Y}_{i}-\overline{Y }\right)}^{2}}}$$

where ${X}_{i}$ and ${Y}_{i}$ denote the individual LFP values at time point $i$, $\overline{X }$ and $\overline{Y }$ are the means over the time window, and $N$ is the total number of data points. The coefficient ranges from − 1 to 1, with 1 indicating identical temporal patterns, 0 signifying no similarity, and − 1 indicating perfectly inverse patterns.

In addition to cross-correlation, phase synchronization between the LFP and the tACS waveform was assessed using a phase coherence metric. During the active tACS period, a sliding window analysis (2.5-s window with 0.1-s steps) was performed. Phase coherence was computed as^89^:

$$Phase\; Coherence= \frac{1}{N}\left|{\sum }_{n=1}^{N}{e}^{i\left({\theta }_{tACS}\left(n\right)-{\theta }_{LFP}\left(n\right)\right)}\right|$$

where ${\theta }_{tACS}\left(n\right)$ and ${\theta }_{LFP}\left(n\right)$ represent the instantaneous phases of the tACS waveform and the LFP at time point $n$, respectively, and $N$ is the number of samples in the window.

At the cellular level, neural entrainment was quantified using the phase-locking value (PLV), which measures the synchronization of neural spiking to the tACS waveform. The PLV is defined as^12^:

$$PLV= \frac{1}{N}\left|{\sum }_{n=1}^{N}{e}^{i{\theta }_{k}}\right|$$

where $N$ is the total number of spikes during the tACS simulation period, and ${\theta }_{k}$ is the phase of the tACS waveform at the *k*th spike. Both the phase coherence metric and PLV range from 0 to 1, with 0 indicating a uniform distribution of phases and 1 indicating perfect synchronization.

For each tACS amplitude and neuron model, spiking phases during the stimulation period were aggregated over 20 simulation runs to compute a single PLV. For instance, under the theta endogenous oscillation, each simulation produced approximately 50 spikes during the active tACS period, resulting in a total of about 1000 spiking phases used to compute a single PLV for each tACS amplitude and neuron model. Additionally, polar histograms were generated to illustrate the distribution of spiking phases relative to the tACS waveform, where 0° corresponds to the waveform’s peak, 180° to its trough, and the preferred phase was determined by the circular mean of the observed phases.

Previous studies have reported that a finite number of samples can bias the PLV when the sample size is insufficient^85,86^. To account for this potential bias, we employed a bias-free, sample-size–independent metric, the pairwise phase consistency (PPC). The PPC is defined as^85^:

$$PPC= \frac{2}{N(N-1)}\sum_{j=1}^{N-1}\sum_{k=j+1}^{N}\mathrm{cos}({\theta }_{j}-{\theta }_{k})$$

using the same notation as in the PLV equation mentioned above. PPC theoretically ranges from –1 to 1, where –1 corresponds to two phases differing by π, 0 to a uniform phase distribution, and 1 to perfect synchronization. For sufficiently large N, the squared PLV converges to the PPC^85,86^.

## Supplementary Information

Below is the link to the electronic supplementary material.

Supplementary Material 1

## Data Availability

The original source code representing human cortical neuron models are available on ModelDB ([https://modeldb.science/241165] (https:/modeldb.science/241165) ). A dataset illustrating the synaptic properties of neuronal populations across the juvenile rat somatosensory cortex is available through the NMC Portal ([https://bbp.epfl.ch/nmc-portal/microcircuit.html] (https:/bbp.epfl.ch/nmc-portal/microcircuit.html)). Although the simulation code is not publicly accessible, it is available from the corresponding author upon appropriate request for academic use.
