# Supplementary material for: Intensity-dependent tACS entrainment effects in a cortical microcircuit: a computational study
Source: Sci Rep. 2026 Jan 31;16:6825. doi: 10.1038/s41598-026-37594-9 (PMC12916757; doi:10.1038/s41598-026-37594-9)
Supplement: Supplementary file 1 — Supplementary Material 1 [file 41598_2026_37594_MOESM1_ESM.docx]

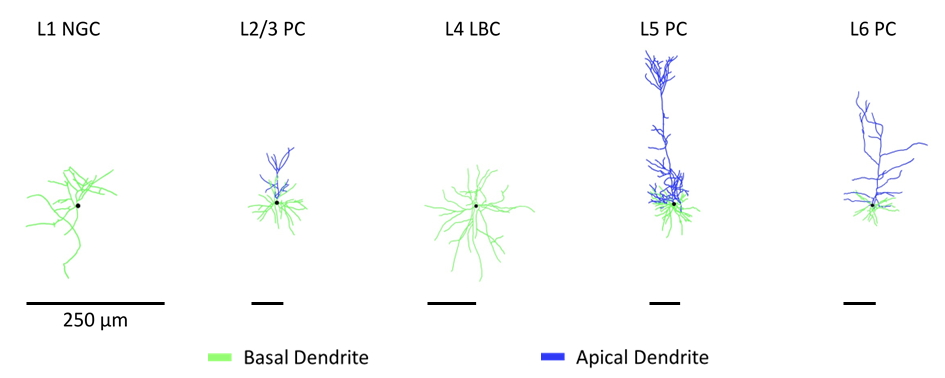


Supplementary Figure S1. Morphologies of basal dendrite and apical dendrite compartments in biophysically realistic human neuron models. The scale bar represents 250 micrometers for each neuron model.


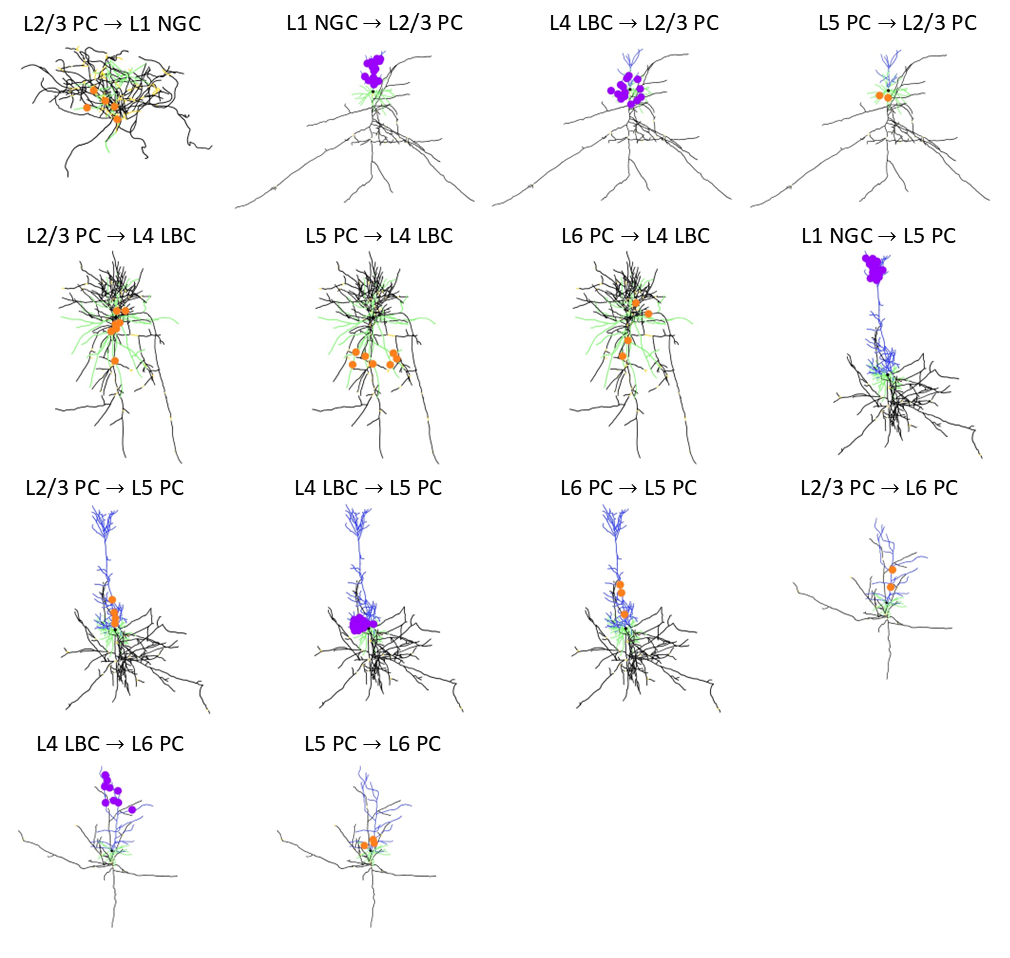


Supplementary Figure S2. Spatial distribution of postsynaptic sites, with presynaptic-to-postsynaptic connections indicated at the top of each panel. Excitatory synaptic sites are marked in orange, while inhibitory synaptic sites are marked in purple.

Supplementary Figure S3. Average power spectral density (PSD) at the time window of active tACS (2.5 – 12.5 s) in the baseline condition for alpha and theta oscillations. 4th order Butterworth bandpass filter with a cutoff frequency of 0.1 and 250 Hz were applied to the raw local field potentials. The gray line denotes 10 Hz for the alpha-band condition and 5 Hz for the theta-band condition.


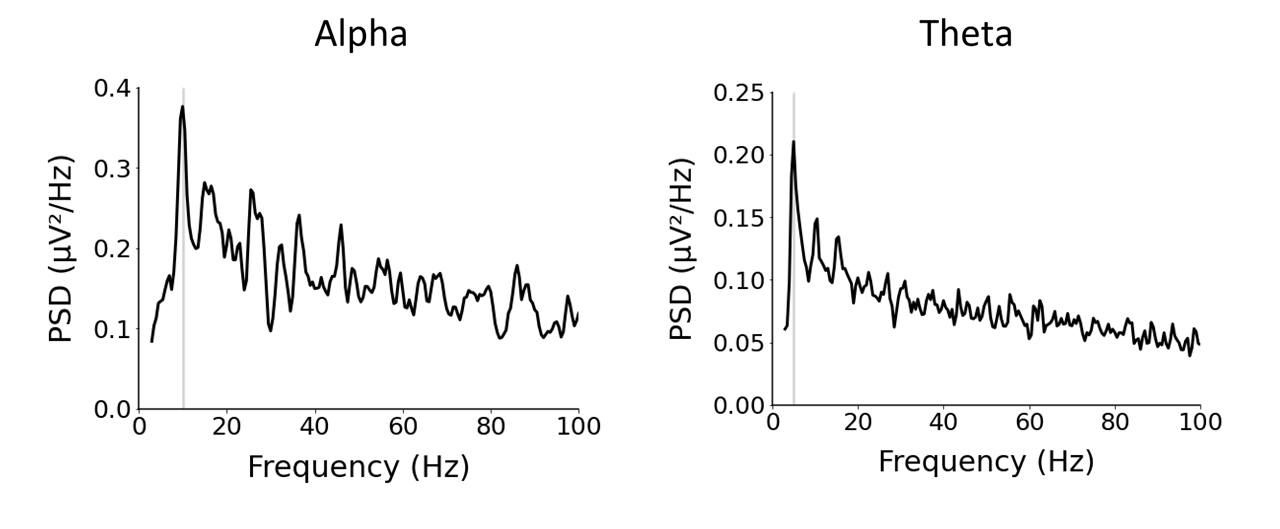

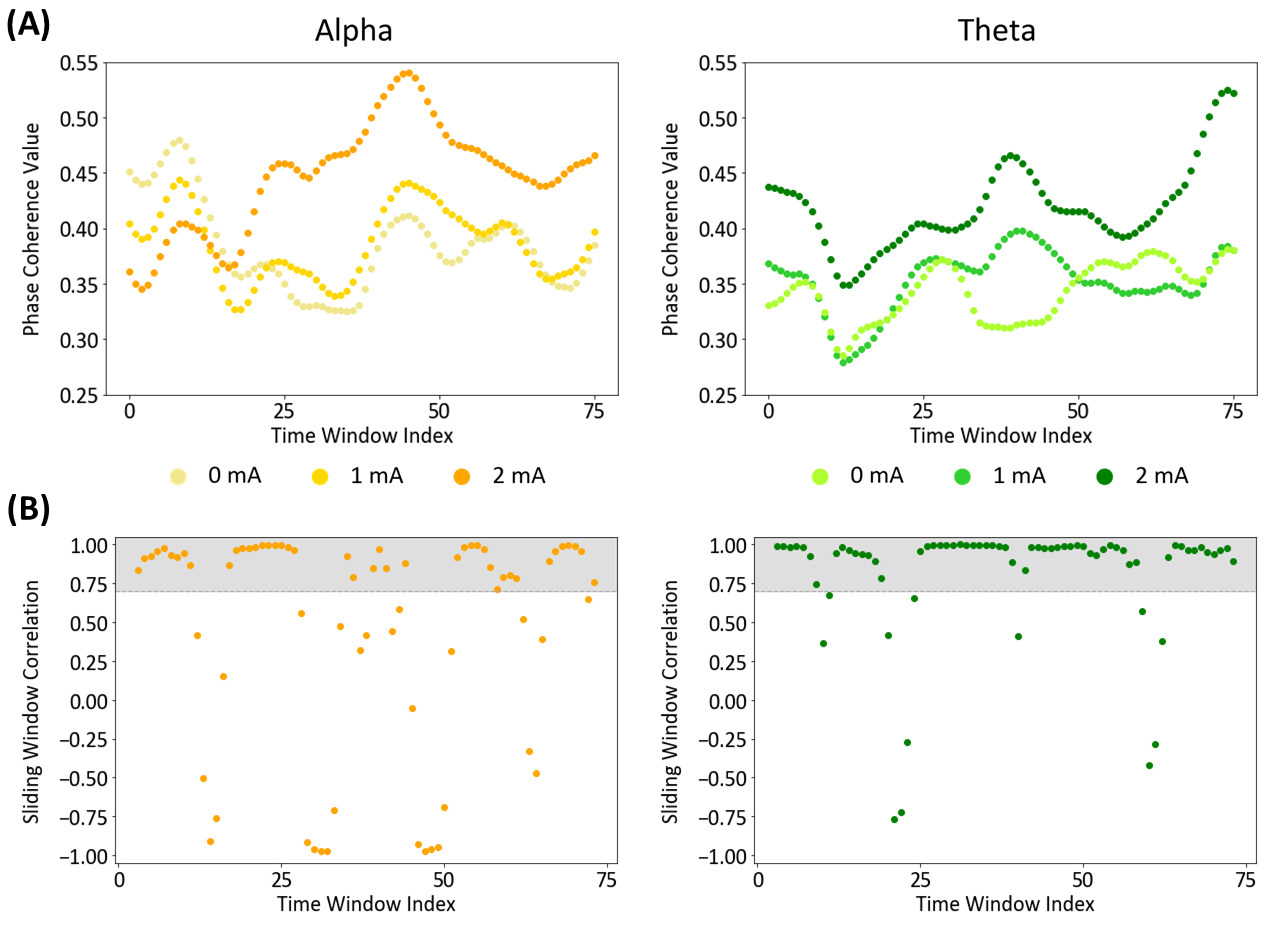


Supplementary Figure S4. (A) Time evolution of averaged phase coherence between LFPs and the tACS waveform in the alpha (left) and theta (right) frequency bands during active stimulation. Alpha and theta oscillations are represented in orange and green, respectively, with darker shades indicating higher stimulation intensities. (B) Sliding window correlations illustrating the relationship between phase coherence differences for 0 mA vs. 2 mA and 0 mA vs. 1 mA conditions in the alpha (left) and theta (right) frequency bands. The sliding window size was set to 6 samples, with time window indicies assigned to the midpoint of each window. Correlation values of 0.7 or higher are highlighted in gray.


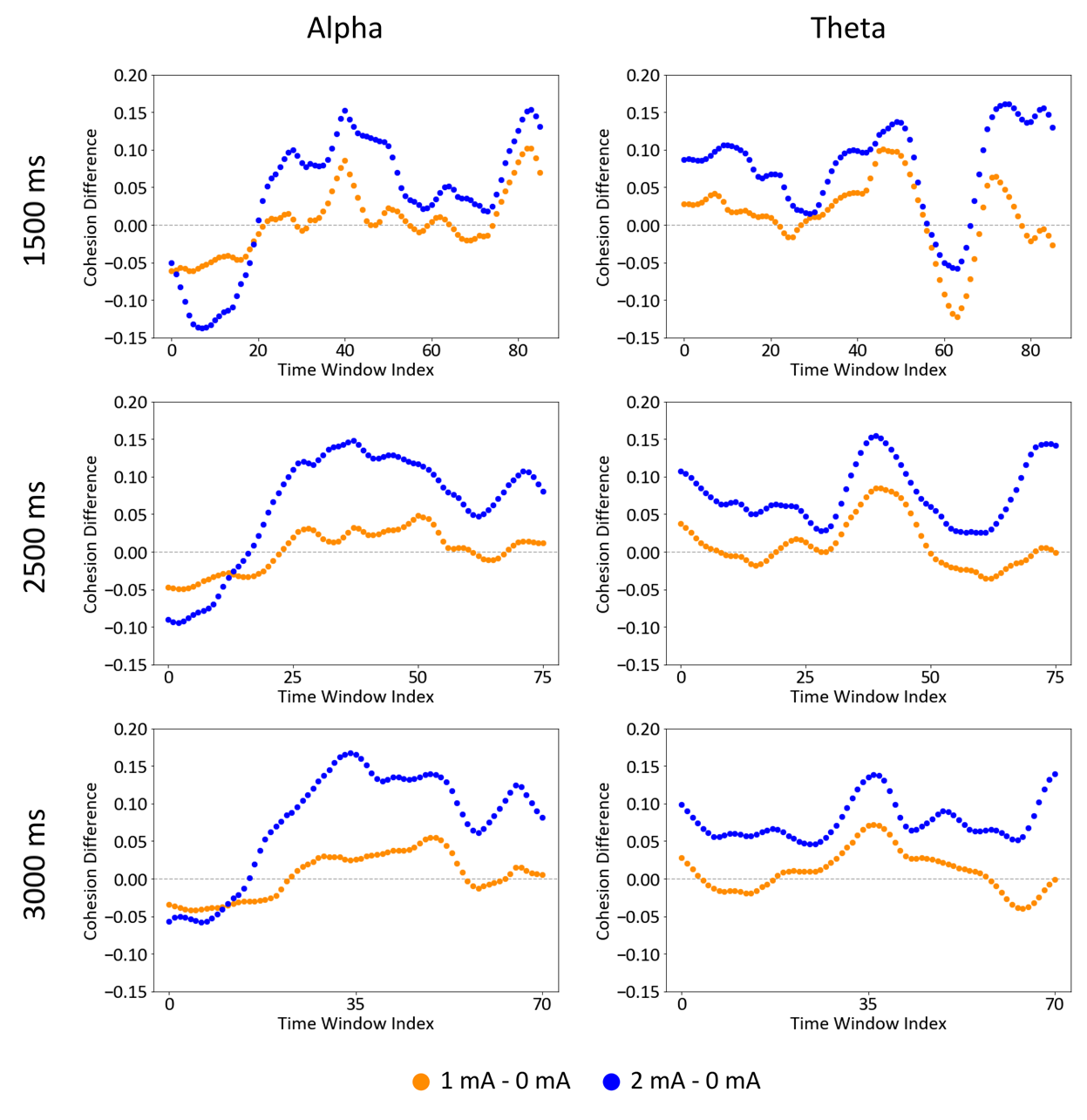


Supplementary Figure S5. Time evolution of averaged phase cohesion difference between LFPs and the tACS waveform in the alpha (left) and theta (right) frequency bands during active tACS simulation. The window length used to compute the phase cohesion values were 1500 ms (top panel), 2500 ms (middle panel), and 3000 ms (bottom panel) with 0.1 second step sizes.

Supplementary Table S1. Phase locking value (PLV) and preferred phase across tACS intensities in the simulated cortical microcircuit. The table shows the PLV and the preferred phase of neuronal spiking relative to the tACS waveform across a range of stimulation intensities (0.1 - 2.0 mA) and the baseline (0 mA). Values are shown for each neuron type—in the order of layer 1 neurogliaform cell (L1 NGC), layer 2/3 pyramidal cell (L2/3 PC), layer 4 large basket cell (L4 LBC), layer 5 pyramidal cell (L5 PC), and layer 6 pyramidal cell (L6 PC)—under both alpha- and theta-band oscillatory conditions, respectively. Additionally, values are represented for microcircuits with full synaptic connectivity (labeled as “O Syn” in the table) and for synaptically disconnected conditions (labeled as “X Syn” in the table) receiving only Poisson-driven inputs. For each condition, spiking phases during the active tACS period were aggregated across 20 independent simulation runs to compute a single PLV and preferred phase. Preferred phase is defined as the circular mean of spiking phases relative to the tACS waveform, where 0° corresponds to the peak and 180° to the trough of the stimulation waveform.

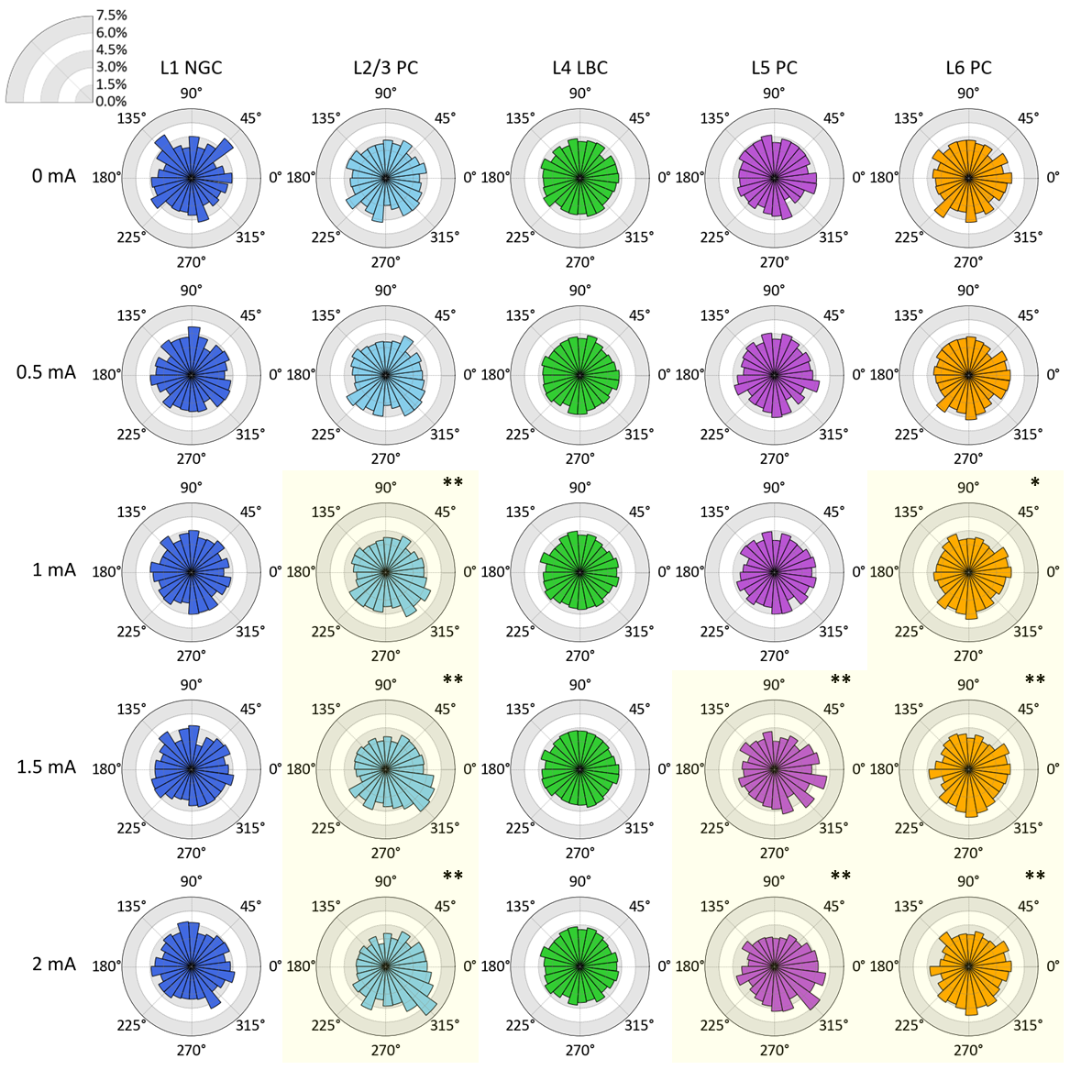


Supplementary Figure S6. Normalized phase histograms of neuronal spiking activity relative to the 10 Hz tACS waveform in a fully connected microcircuit. The neuron models were designed to reflect endogenous alpha oscillations. The total number of spikes during the tACS period was normalized to 1. The legend in the upper left corner indicates the percentage of spikes in each bin relative to the total number of spikes. Statistical significance of non-uniform phase distributions was assessed using the Rayleigh test, with significance levels indicated as * *p* < 0.05 and ** *p* < 0.005 in the upper right corner of each phase histogram.


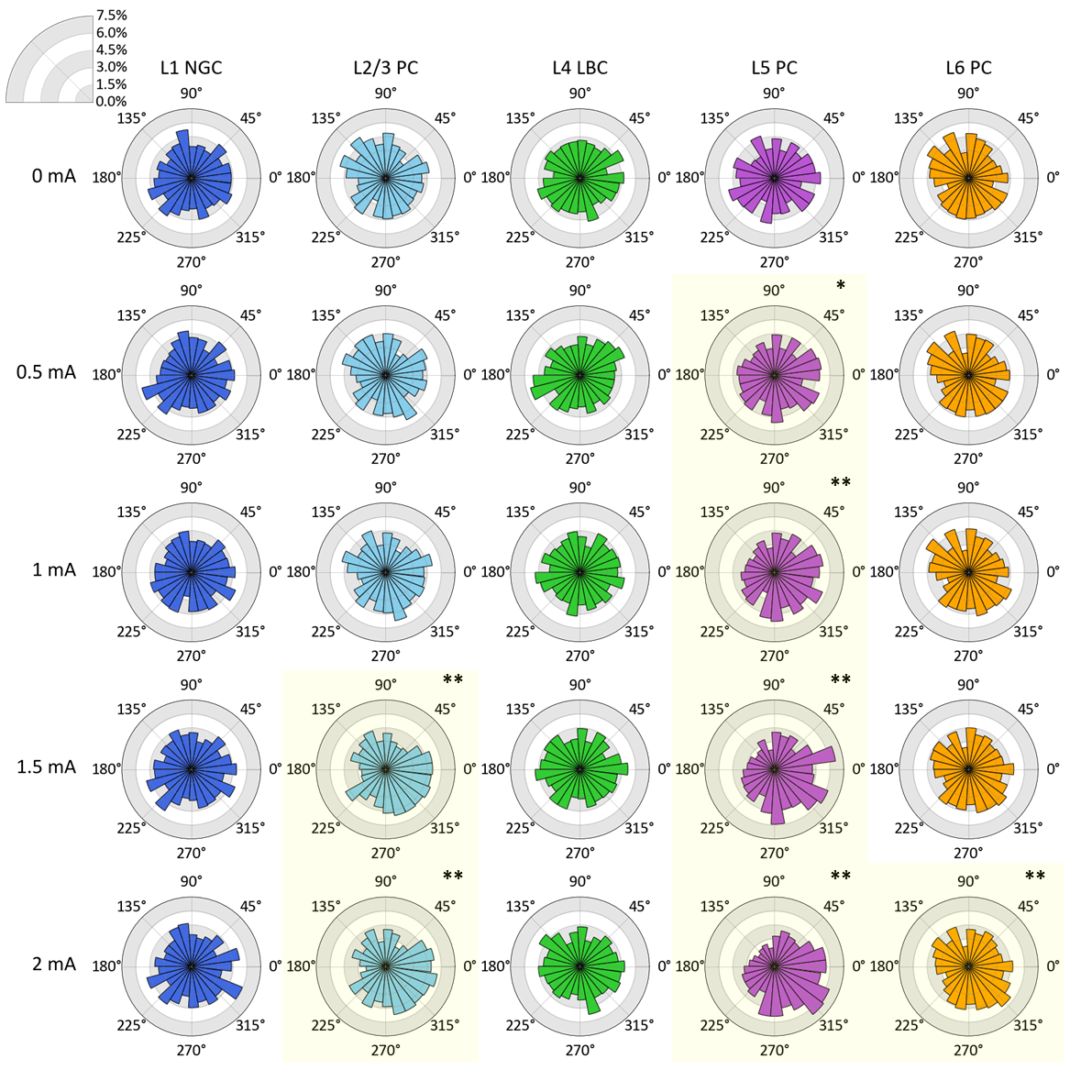


Supplementary Figure S7. Normalized phase histograms of neuronal spiking activity relative to the 5 Hz tACS waveform in a fully connected microcircuit. The neuron models were designed to reflect endogenous theta oscillations. The total number of spikes during the tACS period was normalized to 1. The legend in the upper left corner indicates the percentage of spikes in each bin relative to the total number of spikes. Statistical significance of non-uniform phase distributions was assessed using the Rayleigh test, with significance levels indicated as * *p* < 0.05 and ** *p* < 0.005 in the upper right corner of each phase histogram.

Supplementary Figure S8. Normalized phase histograms of neuronal spiking activity relative to the tACS waveform in a fully connected microcircuit. From top to the bottom panel, 5 Hz, 8 Hz, 10 Hz, and 12 Hz 1 mA tACS was applied to the microcircuit, respectively. The neuron models were designed to reflect endogenous alpha oscillations and the total number of spikes during the tACS period was normalized to 1. The legend in the upper left corner indicates the percentage of spikes in each bin relative to the total number of spikes. Statistical significance of non-uniform phase distributions was assessed using the Rayleigh test, with significance levels indicated as * *p* < 0.05 and ** *p* < 0.005 in the upper right corner of each phase histogram. Phase locking values corresponding to * *p* < 0.05 ranged from 0.04 to 0.05 and ** *p* < 0.005 ranged from approximately from 0.06 to 0.08.


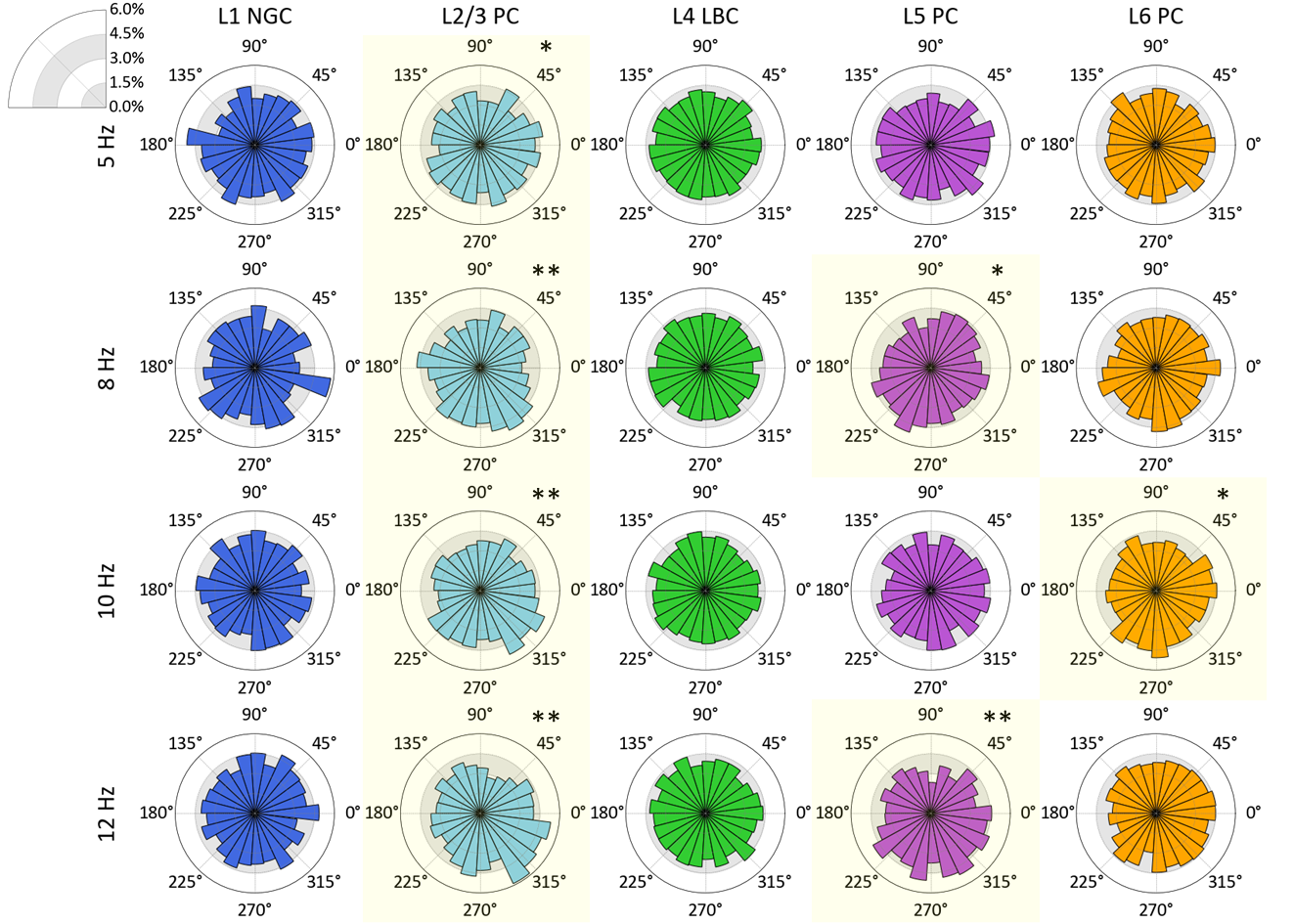


Supplementary Figure S9. Normalized phase histograms of neural spiking activity in a fully connected microcircuit at the baseline conditions (with no stimulations). The neuron models were designed to reflect endogenous alpha oscillations and the total number of spikes during the tACS period was normalized to 1. The legend in the upper left corner indicates the percentage of spikes in each bin relative to the total number of spikes. Using the Rayleigh test, all of the phase histograms displayed *p* > 0.05, with phase locking values lower than 0.04.


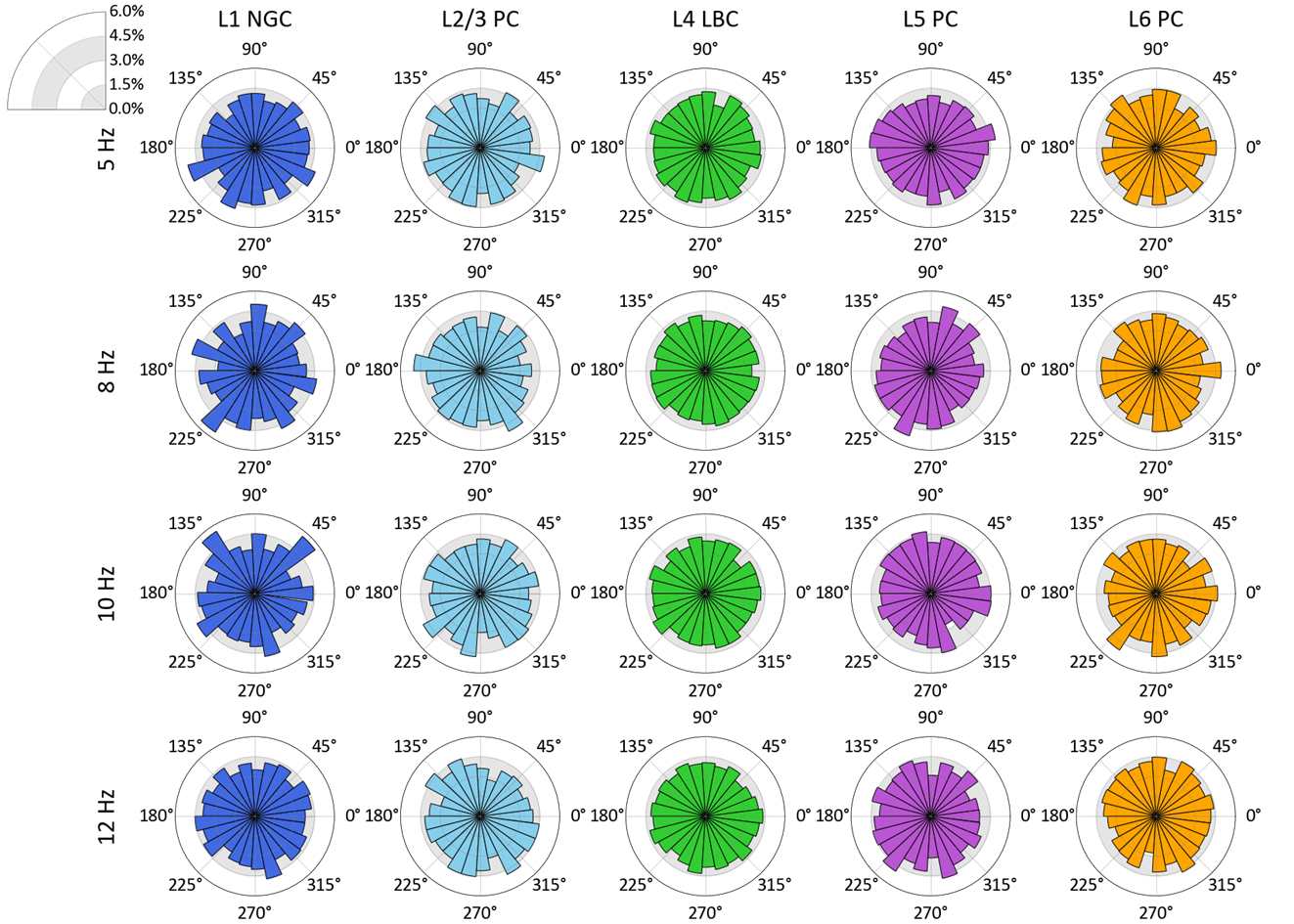

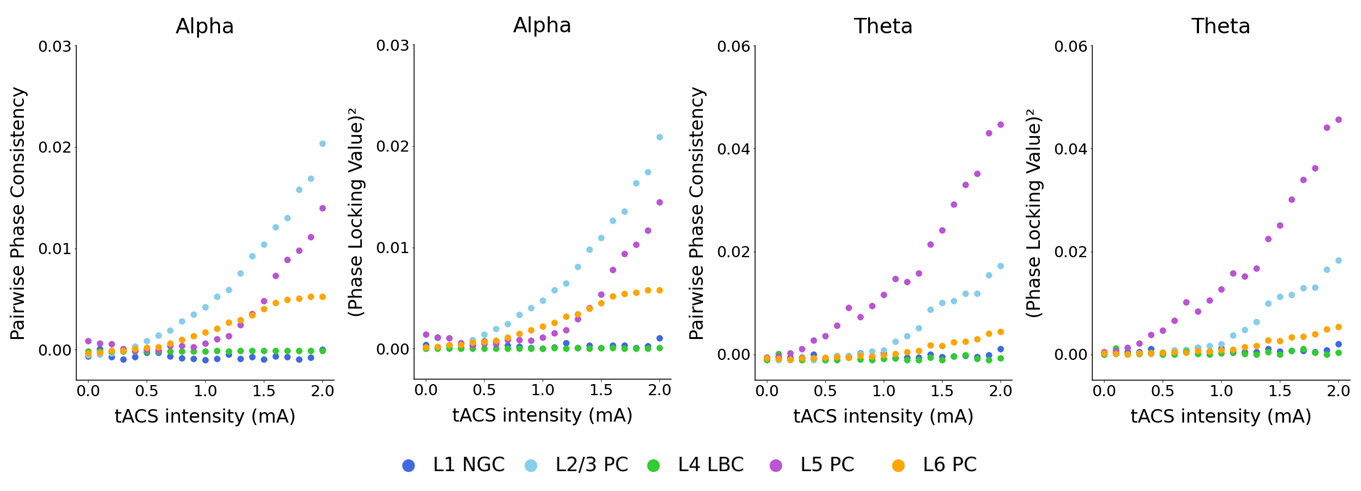


Supplementary Figure S10. Pairwise phase consistency and squared value of the phase locking value corresponding to tACS intensity in alpha (left) and theta (right) oscillations.


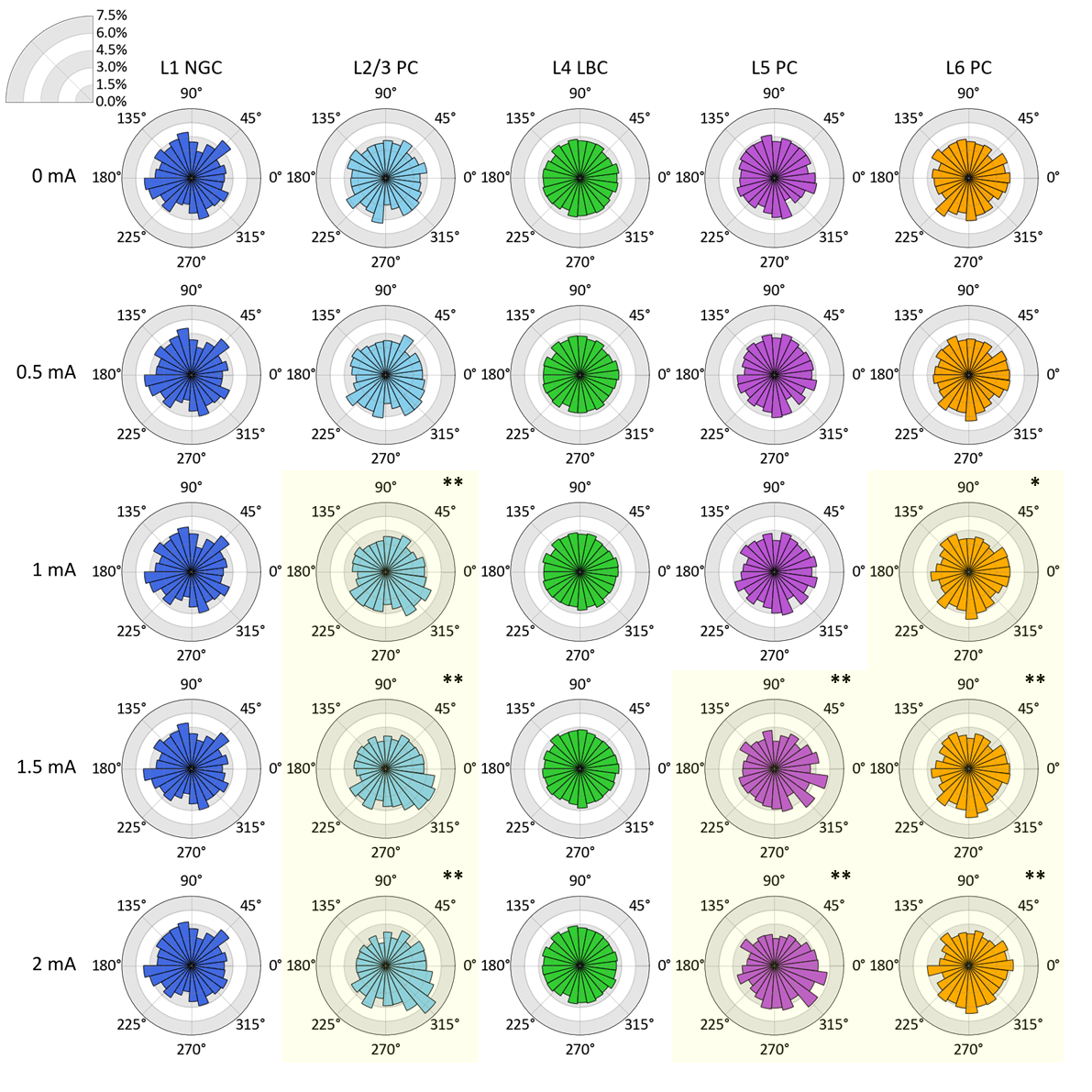


Supplementary Figure S11. Normalized phase histograms of neuronal spiking activity relative to the 10 Hz tACS waveform in a synaptically disconnected microcircuit. The neuron models were designed to reflect endogenous alpha oscillations. The total number of spikes during the tACS period was normalized to 1. The legend in the upper left corner indicates the percentage of spikes in each bin relative to the total number of spikes. Statistical significance of non-uniform phase distributions was assessed using the Rayleigh test, with significance levels denoted as * *p* < 0.05 and ** *p* < 0.005 in the upper right corner of each phase histogram.


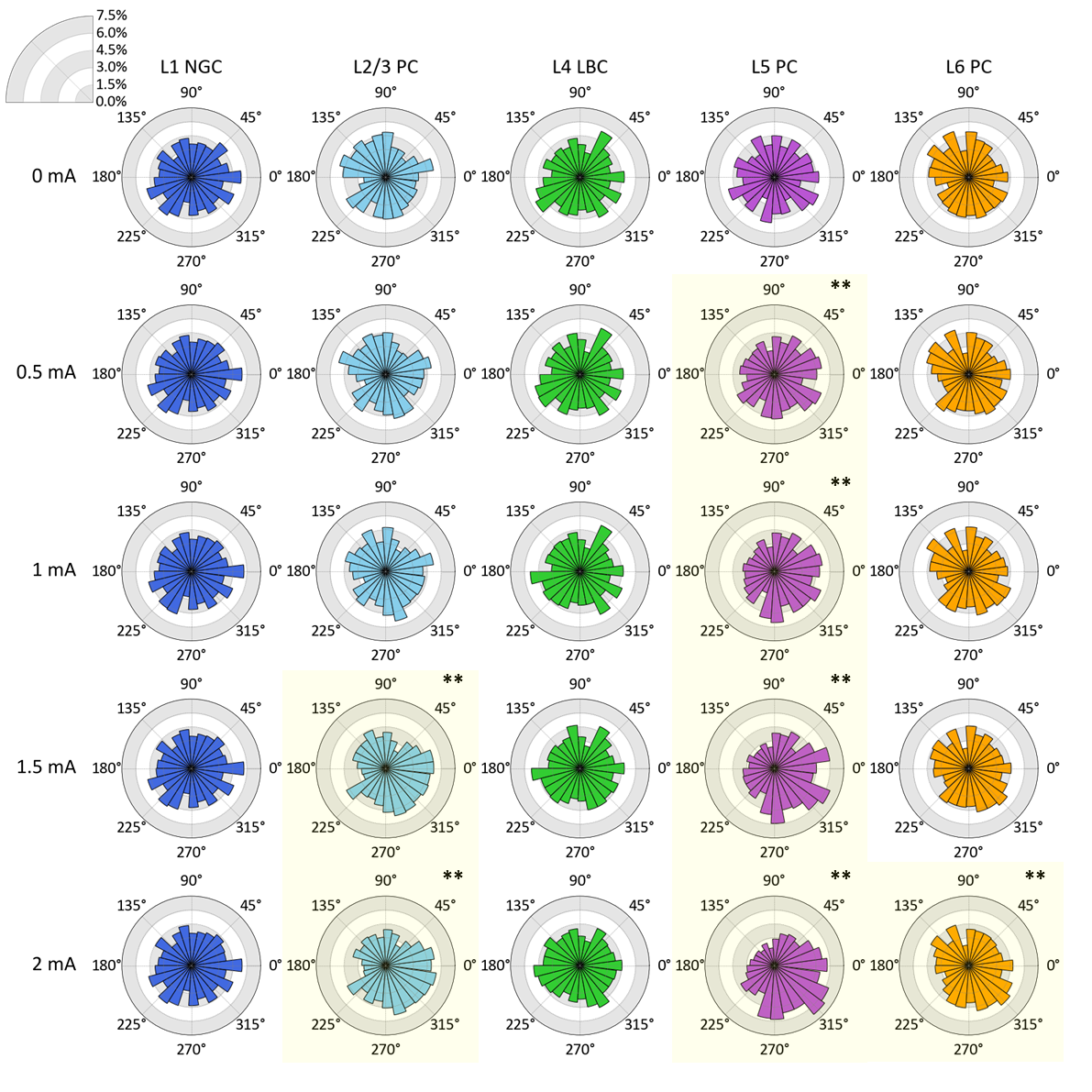


Supplementary Figure S12. Normalized phase histograms of neuronal spiking activity relative to the 5 Hz tACS waveform in a synaptically disconnected microcircuit. The neuron models were designed to reflect endogenous theta oscillations. The total number of spikes during the tACS period was normalized to 1. The legend in the upper left corner indicates the percentage of spikes in each bin relative to the total number of spikes. Statistical significance of non-uniform phase distributions was assessed using the Rayleigh test, with significance levels denoted as * *p* < 0.05 and ** *p* < 0.005 in the upper right corner of each phase histogram.
